# ABE8e with Polycistronic tRNA-gRNA Expression Cassette Sig-Nificantly Improves Adenine Base Editing Efficiency in *Nicotiana benthamiana*

**DOI:** 10.3390/ijms22115663

**Published:** 2021-05-26

**Authors:** Zupeng Wang, Xiaoying Liu, Xiaodong Xie, Lei Deng, Hao Zheng, Hui Pan, Dawei Li, Li Li, Caihong Zhong

**Affiliations:** 1 Key Laboratory of Plant Germplasm Enhancement and Specialty Agriculture, Wuhan Botanical Garden, Chinese Academy of Sciences, Wuhan 430074, China; liuxiaoying17@mails.ucas.ac.cn (X.L.); tycxxd02070624@163.com (X.X.); chinese_denglei@foxmail.com (L.D.); zhenghao210524@163.com (H.Z.); panhui@wbgcas.cn (H.P.); lidawei@wbgcas.cn (D.L.); lily19851205@aliyun.com (L.L.); 2 Innovative Academy of Seed Design, Chinese Academy of Sciences, Beijing 100101, China; 3 Engineering Laboratory for Kiwifruit Industrial Technology, Chinese Academy of Sciences, Wuhan 430074, China

**Keywords:** CRISPR, base editor, ABE, ABE8e, STU, PTG

## Abstract

Adenine base editor containing TadA8e (ABE8e) has been reported in rice. However, the application of ABE8e in other plant species has not been described, and the comparison between ABE8e and ABE7.10, which is widely used in plants, has also been poorly studied. Here, we developed the ABE8e with the polycistronic tRNA-gRNA expression cassette (PTG-ABE8e) and PTG-ABE7.10 and compared their A-to-G editing efficiencies using both transient and stable transformation in the allotetraploid *Nicotiana benthamiana*. We found that the editing efficiency of PTG-ABE8e was significantly higher than that of PTG-ABE7.10, indicating that ABE8e was more efficient for A-to-G conversion in *N. benthamiana*. We further optimized the ABE8e editing efficiency by changing the sgRNA expression cassette and demonstrated that both PTG and single transcript unit (STU) enhanced ABE8e efficiency for A-to-G conversion in *N. benthamiana*. We also estimated the potential off-target effect of PTG-ABE8e at potential off-targeting sites predicted using an online tool in transgenic plants, and no off-target editing event was found for potential off-targeting sites selected, indicating that ABE8e could specifically facilitate A-to-G conversion. Our results showed that ABE8e with PTG structure was more suitable for A-to-G conversion in *N. benthamiana* and provided valuable clues for optimizing ABE tools in other plants.

## 1. Introduction

Single nucleotide polymorphisms (SNPs) are significantly associated with the diversity of agriculture traits of crops, and SNPs are the potential target site for molecular breeding [1]. Hence, precise tools inducing single base substitutions are crucial for precise molecular breeding [2]. CRISPR/Cas-based genome editing tools widely used in targeted genome modifications usually induce small indels [2]. The precise genome editing via the homology-directed repair pathway (HDR) can facilitate single base mutations or insert desired sequences into the target sites, requiring a donor template [3]. However, the low efficiency of HDR and the delivery of donor template is a constraint to its application in plants [2,4]. Newly developed base editors (BEs) including cytosine base editor (CBE) and adenine base editor (ABE) do not require the generation of double-strand breaks (DSBs) and DNA templates for DNA repair to induce base substitutions [2,4]. CBE contains the cytidine deaminase, the Cas9 nickase (nCas9) or deactivated Cas9 (dCas9), and the uracil glycosylase inhibitor (UGI), which can convert cytidine (C) to thymidine (T) (or G to A) at specific genomic sites [2,4]. The ABE tool converts A to G (or T to C). It consists of the nCas9 fused with an evolved adenine deaminase TadA from *E. coli*. It has been used in rice and other crops [4]. In plants, the ABE7.10, which harbors a TadA variant, TadA 7.10, is the most widely applied BE to specific A-to-G conversions [2,4]. The ABE7.10 has been used in *Oryza sativa*, *Triticum aestivum*, *Arabidopsis thaliana*, and *Brassica napus* [5,6,7,8,9,10,11]. 

Although the efficiency of ABE 7.10 is significantly higher than that of precise genome editing mediated by HDR, its efficiency still needs to improve and varies significantly across different targets, which hampers their use in precise plant genome engineering and crop breeding [9,10]. The previous study has compared the effects of sgRNA structures and sgRNA expression system in rice and found that sgRNA induces the highest A-to-G conversion [10]. Moreover, the efficiency of ABE7.10 is enhanced by simplifying the structure of ABE 7.10 in rice [9]. Recently, a new ABE (ABE8e) containing a new adenine deaminase TadA variant TadA-8e was used to verify that ABE8e catalyzes deamination up to 1100 times faster than the ABE7.10 [12,13]. The efficiency of ABE8e is significantly improved in human cells, especially at poorly edited targets with ABE7.10, which could provide a useful tool for broadening adenine base editing capability [12]. While we prepared this manuscript, the ABE8e has been reported in rice [14,15,16]. However, the comparison between ABE7.10 and ABE8e and the effects of the sgRNA expression system on ABE8e have not been investigated. In this study, we compared the efficiencies of ABE7.10 and ABE8e using four targets in a model allotetraploid tobacco (*Nicotiana benthamiana*). Next, we estimated the effects of different sgRNA expression cassettes on ABE8e.

## 2. Results

### 2.1. ABE8e Induced Higher A-to-G Conversion than ABE7.10

**Figure 1:**
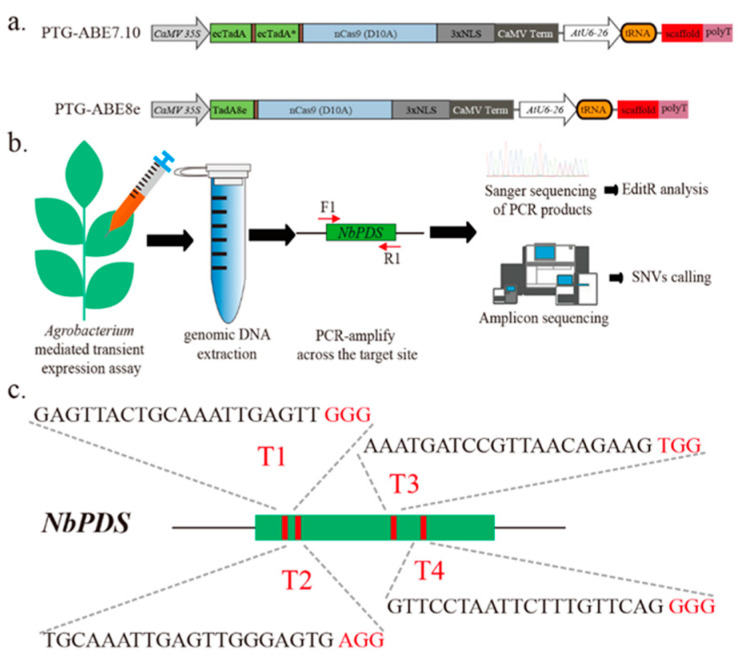
Overview of A-to-G base editing by different ABEs in *N. benthamiana*. (**a**) Schematic depicting the PTG-ABE7.10 and PTG-ABE8e structure. NLS, nuclear localization sequence. (**b**) Assay scheme of agrobacterium-mediated transient expression and base-editing identification. F1/R1 indicates binding sites of the primers used for species-specific PCR amplification. (**c**) Schematic illustrating the four sgRNAs targeting the NbPDS gene. Red letters indicate the PAM sequence of gRNA.

To develop the ABE8e variant codon-optimized for plants, we first used an ABE8e vector PTG-ABE8e, which combined polycistronic tRNA-gRNA expression cassette (PTG) (Figure 1a). We also constructed PTG-ABE7.10 using the method described in Li et al. and used it as control (Figure 1a) [10]. We designed four gRNAs targeting the *NbPDS* gene (T1-T4) and conducted *agrobacterium*-mediated transient expression assays to examine the base editing of ABE7.10 and ABE8e in *Nicotiana benthamiana* (Figure 1b,c). The leaves were harvested three days post-infiltration, and the genomic DNA was extracted for base-editing identification (Figure 1b).

**Figure 2:**
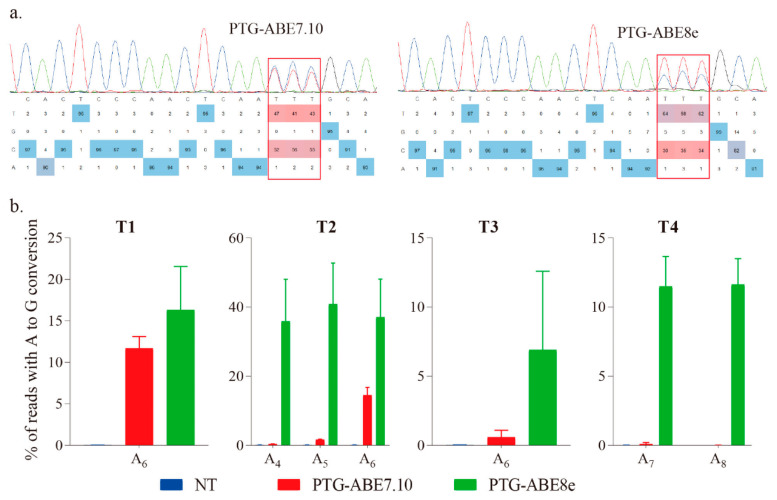
Estimation of A-to-G base editing induced by PTG-ABE7.10 and PTG-ABE8e in *N. benthamiana*. (**a**) Sanger sequencing chromatograms of the T2 target for PTG-ABE7.10 and PTG-ABE8e. The sequencing chromatograms show the results sequenced from the reverse direction. Red squares indicate the edited bases induced by ABEs. (**b**) Frequencies of A-to-G conversions induced by PTG-ABE7.10 and PTG-ABE8e at four target sites based on amplicon deep sequencing results. Frequencies (mean ± SEM) were calculated from three independent experiments (*n* = 3). NT, no-treatment.

The targeted region was amplified using species-specific primers (F1 and R1), and the PCR products were sent for Sanger sequencing and base editing identification using EditR [17]. The results showed that both ABE7.10 and ABE8e could successfully induce precise A-to-G conversion (Figure 2a). To accurately evaluate the editing efficiency of the four ABEs, we conducted the targeted amplicon deep sequencing for the targeted regions, and the data were analyzed using CRISPResso2 with batch mode [18]. The ABE8e harbored higher editing efficiency for all four targets than that of ABE7.10 (Figure 2b). The editing efficiency of PTG-ABE8e (range: 1.32–41.77%, mean: 22.89%) was 5.54-fold higher than that of PTG-ABE7.10 (range: 0–15.92%, mean: 4.13%), indicating that PTG-ABE8e is more efficient than PTG-ABE7.10 in *N. benthamiana* plants (Figure 2b). The PTG-ABE8e also had the highest on-target indel efficiency, but percentages of on-target indels of both ABE8e and ABE7.10 were extremely low (<0.06%) (Figure S1).

### 2.2. SgRNA Expression Cassettes Significantly Affected Editing Efficiencies of ABE8e

**Figure 3:**
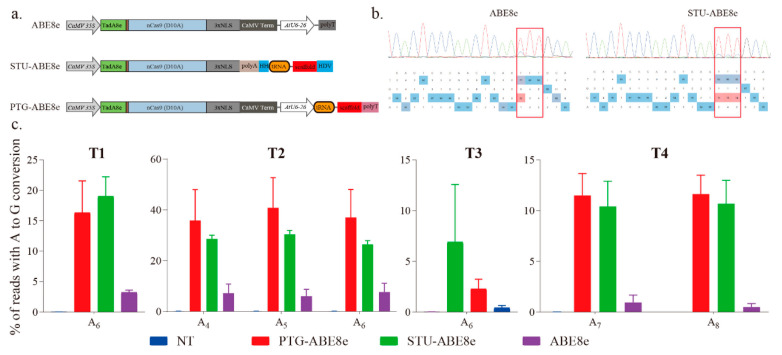
Estimating A-to-G base editing induced by ABE8e variants harboring different sgRNA expression cassettes in *N. benthamiana*. (**a**) Schematic depicting ABE8e variants structure. NLS, nuclear localization sequence; HH, hammerhead ribozyme; HDV, hepatitis delta virus ribozyme. (**b**) Sanger sequencing chromatograms of the T2 target for ABE8e variants. The sequencing chromatograms show the results sequenced from the reverse direction. Red squares indicate the edited bases induced by ABE8e variants. (**c**) Frequencies of A-to-G conversions induced by ABE8e variants at four target sites based on amplicon deep sequencing results. Frequencies (mean ± SEM) were calculated from three independent experiments (*n* = 3). NT, no-treatment.

We further used three sgRNA expression cassettes to investigate the effects of sgRNA expression cassette on the editing efficiency of ABE8e (Figure 3a). The expression cassette of ABE8e is a regular expression cassette used in previous studies [14,15,19]. The cassette of STU-ABE8e combined the sgRNA and Cas9 into a single transcript unit (STU) (Figure 3a). The four targets described above were applied to compare different sgRNA expression cassettes (Figure 1c). The sequencing chromatograms of corresponding PCR products indicated that all the three ABE8e constructions successfully induced A-to-G conversions (Figure 2a and Figure 3b). Further, editing efficiencies of three ABE8e for all four targets were estimated using the deep amplicon sequencing. Editing efficiencies of both PTG-ABE8e and STU-ABE8e were significantly higher than that of ABE8e (Figure 3c). The editing efficiency of PTG-ABE8e was slightly higher than that of STU-ABE8e (Figure 3c). Those results verified that both PTG and STU expression cassette could enhance the editing efficiency of ABE8e in *N. benthamiana*. The on-target indel frequencies induced by three ABE8e constructions in the four targets were lower than 0.1%, indicating that ABE8e could facilitate precise A-to-G conversion with low byproducts (Figure S2). 

**Figure 4:**
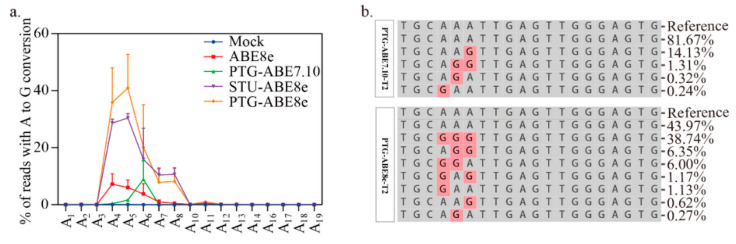
Editing window and editing outcome comparison between PTG-ABE7.10 and PTG-ABE8e. (**a**) The deamination window for all ABE variants inducing A-to-G conversion. NT, no-treatment. (**b**) The allelic outcome of the T2 site edited by PTG-ABE8e and PTG-ABE7.10.

Moreover, we compared editing windows of ABE8e and ABE7.10. The editing window for ABE8e was identified in previous work (Figure 4a). It ranges from protospacer positions 4 to 8 (Figure 4a). For the four targets tested, the editing efficiency of PTG-ABE8e at different positions (A_4_: 18.35, A_5_: 28.10, A_6_: 25.39, A_7_: 25.98, A_8_:1.31) was significantly higher than that of PTG-ABE7.10 (A_4_: 0.37, A_5_: 1.65, A_6_: 8.93, A_7_: 0.09, A_8_: 0.01) (Figure 4a). Next, we compared the editing outcomes of PTG-ABE8e and PTG-ABE7.10. Unlike PTG-ABE7.10, PTG-ABE8e mainly induced multiple As conversions at the target region, which showed that ABE8e had higher deamination activity (Figure 4b). In summary, sgRNA expression cassettes significantly affected the editing efficiency of ABE8e in *N. benthamiana*.

### 2.3. ABE8e Induced Higher A-to-G Conversion than ABE7.10 in Transgenic Plantlets

To estimate whether ABE8e and ABE7.10 can induce A-to-G conversion in transgenic plantlets of *N. benthamiana*, we conducted stable transformations using T1 and T2 targets (Figure 1c). The genomic DNA was extracted from transgenic plantlets regenerated and used for PCR templates for specific amplification. PCR products were subjected to cloning and sequencing, and 20 positive clones for each sample were checked for editing efficiency estimation. Our results indicated that both PTG-ABE7.10 and PTG-ABE8e could successfully facilitate A-to-G conversion in transgenic plants, and the editing efficiency of PTG-ABE8e was higher than that of PTG-ABE7.10 (Table 1). For the T1 target, 48 and 42 transgenic plants were regenerated for PTG-ABE7.10 and PTG-ABE8e, respectively, and 10 (20.83%) and 13 (30.95%) transgenic plants harbored A-to-G conversions for PTG-ABE7.10 and PTG-ABE8e, respectively (Table 1). For the T2 target, 5 out of 32 (15.63%) and 28 out of 46 (60.87%) transgenic plants contained A-to-G conversions for PTG-ABE7.10 and PTG-ABE8e, respectively (Table 1). All edited transgenic plants for PTG-ABE7.10 were heterozygous at T1 and T2 targets (Table 1), while 15.38% (2 out of 13) and 25% (7 out of 28) edited transgenic plants for PTG-ABE8e were homozygous at T1 and T2, respectively (Table 1).

### 2.4. Off-Target Analysis

To estimate the off-target effect of PTG-ABE8e, we used the online tool CRISPOR (http://crispor.tefor.net/, accessed on 12 December 2019) to predict the potential off-target sites of T1 and T2 targets, and nine putative off-target sites were identified for further analysis (Table 2). All transgenic plants regenerated above were used for estimation of the off-target effect (Table 1). The specific amplicons amplified from genomic DNA extracted from transgenic plants were cloned and sequenced. No A-to-G conversion was found in any putative off-target sites in all transgenic plants selected (Table 2). Those results indicated that PTG-ABE8e could specifically facilitate A-to-G conversions.

## 3. Discussion

Base editors have been applied for precise plant genome modification, including rice, cotton, *Triticum aestivum*, *Arabidopsis thaliana*, and *Brassica napus* [1,2,20,21,22]. In human cells, the editing efficiency of ABE8e is significantly higher than that of ABE7.10 [12]. ABE8e also successfully applied for base editing in rice [15,16]. However, the application of ABE8e, and comparison between ABE8e and ABE7.10, have not been reported in other plant species. In this study, we successfully applied ABE8e to induce A-to-G conversion in model allotetraploid *N. benthamiana* and compared editing efficiencies of ABE8e and ABE7.10 (Figure 1). Our results showed that the editing efficiency of ABE8e was about 5.54-fold for that of ABE7.10, indicating that ABE8e was more efficient than ABE7.10 in *N. benthamiana* (Figure 1).

Previous research verified that both sgRNA expression cassette and sgRNA structure significantly affected the editing efficiency of the ABE7.10 tool in rice [10]. In this study, we compared the effects of three sgRNA expression cassettes on the editing efficiency of ABE8e in *N. benthamiana*. Comparing with the regular sgRNA expression cassette, both STU and PTG cassettes showed higher efficiencies (Figure 2). Those results indicated that STU and PTG cassettes enhanced the base editing efficiency of ABE8e in *N. benthamiana*. We also verified editing efficiencies of PTG-ABE8e and PTG-ABE7.10 using the stable transformation method in *N. benthamiana* (Table 1). Consistent with the transient transformation results, the editing efficiency of PTG-ABE8e was higher than that of PTG-ABE7.10 in transgenic plants (Table 1). Based on analysis of potential off-targeting on the limited selection of calculated most likely genomic targets, the absence of significant off-targeting indicated that PTG-ABE8e could specifically induce precise A-to-G conversion in *N. benthamiana*. According to the results of previous studies, codon optimization can improve the editing efficiency of CRISPR/Cas9 and base editors [2]. Therefore, we speculate that the editing efficiency of ABE8e can be further improved through codon optimization. Previous researches verified that the PTG cassette increases the editing efficiency of CRISPR/Cas9 and base editors in plants and other species, especially for multiple genome editing [2,4,23]. Moreover, the tRNA sequence in the PTG cassette can act as an enhancer to increase the expression level of sgRNA and enhance the efficiency of gene editing [2,4].

In summary, we designed and optimized new ABE variants using Tad8e. All ABE8e variants successfully facilitated A-to-G conversion in endogenous sites of the *N. benthamiana* genome. Our results illustrated that the base editing efficiency of PTG-ABE8e was significantly higher than that of PTG-ABE7.10 in *N. benthamiana*. We also found that the PTG cassette induced the highest base editing efficiency. To sum up, our results showed that ABE8e is a more efficient adenine base editor, and its high efficiency could enhance the precise genome modification in plants and genetic improvement.

## 4. Materials and Methods

### 4.1. Plasmid Construction

The sequences of TadA8e and TadA7.10 with a 96-bp linker were codon-optimized using JCat (http://www.jcat.de/, accessed on 12 September 2019) and commercially synthesized (Genewiz, Suzhou, China). All ABE constructs were modified from the vector pPTG-sgRNA-Cas9-U6-1 [24]. The fragment containing nCas9-3xNLS was amplified and replaced the segment between the BamHI site and the SpeI site in the vector pPTG-sgRNA-Cas9-U6-1 forming the vector PTG-nCas9. The TadA8e coupling with the linker was amplified and inserted into the vector PTG-nCas9 forming the PTG-ABE8e construct. The TadA-TadA7.10- linear fragment was inserted into the vector PTG-nCas9 generating the PTG-ABE7.10 construct. The fragment containing AtU6-26 promoter, two BsaI sites, gRNA scaffold, and polyT replaced the sequences between the two AscI sites in the vector PTG-ABE8e forming ABE8e. The fragment containing hammerhead (HH) ribozyme, two BsaI sites, gRNA scaffold, and hepatitis delta virus (HDV) ribozyme was synthesized and inserted into the vector PTG-ABE8e forming STU-ABE8e-inter construct. The STU-ABE8e-inter vector was digested by AscI and ligated using T4 DNA ligase generating STU-ABE8e construct.

All four sgRNAs targeting the *NbPDS* gene were designed using the online tool CRISPOR (http://crispor.tefor.net/, accessed on 12 December 2019) (Table S1), and the corresponding primers were designed using in-house Perl scripts, and then commercially synthesized (TsingKe, Beijing, China) (Table S2). According to the protocol described in our previous study [24], each sgRNA was cloned into the ABE binary vectors forming final constructs used for transformation arrays.

### 4.2. Transient Expression Assay in N. benthamiana

Plantlets of *N. benthamiana* were grown in a growth chamber at 26 °C with a 16 h photoperiod. The transformation protocol of *N. benthamiana* was modified from the procedure described in Shan et al. [25]. *Agrobacterium tumefaciens* strain EHA105 harboring the final ABE binary construct was cultured on Lysogeny broth (LB) medium supplemented with 50 mg L^−1^ Kanamycin at 28 °C. The EHA105 cells were then pelleted and resuspended in MES buffer in the dark for 4 h at room temperature (RT) before infiltration. Leaves of 3–4 weeks *N. benthamiana* plants were infiltrated with *Agrobacterium tumefaciens* EHA105 strains carrying the final binary vectors with OD600 = 0.4. The leaves were harvested three days post infiltration, and the genomic DNA was extracted for base-editing identification according to the instruction of the DNAsecure plant kit (TIANGEN, Beijing, China).

### 4.3. Deep Amplicon Sequencing and Data Analysis

The genomic DNA extracted above was used as the PCR template. The site-specific primer was designed and used for the first-round PCR using the TransStart^®^ FastPfu DNA Polymerase according to the instruction (TransGen Biotech, Beijing, China). Further, the PCR products of the first-round added the forward and reverse barcodes for library construction. The PCR product of each sample was sequenced commercially using the Illumina HiSeq 2500 platform (Lc-Bio Technologies, Hangzhou, China). The clean reads number of each sample ranged from 54,000 to 88,000. The amplicon sequencing was set for three replicates of each transient expression array. The on-target base editing efficiencies were analyzed using CRISPResso2 with the batch mode with default parameters.

### 4.4. PCR Product Cloning and Sanger Sequencing

Amplicom products of samples transformed with ABE binary vectors harboring T2 target were cloned into the pClone007 vector using a pClone007 simple vector kit (TsingKe, Beijing, China). The ligated products were transformed into *Escherichia coli* strain DH5a cells, and ten positive clones were selected for Sanger sequencing. The DNAMAN software (v4.0; Lynnon Corporation, Vaudreuil, Quebec, Canada) was used for alignment analysis.

### 4.5. Agrobacterium-Mediated Stable Transformation in N. benthamiana

The stable transformation of *N. benthamiana* was conducted according to the protocol described previously [25]. The concentration of kanamycin selection was 100 mg L^−1^. Genomic DNA was extracted from transgene plants regenerated, and amplicons were amplified using specific primers, and cloned into the pClone007 vector using a pClone007 simple vector kit (TsingKe, Beijing, China). The ligated products were transformed into *Escherichia coli* strain DH5a cells, and 30 positive clones were selected for Sanger sequencing. The DNAMAN software (v4.0; Lynnon Corporation, Canada) was used for alignment analysis.

### 4.6. Statistical Analysis

All the experiments in this study consisted of three replicates. Uncertainties in the reported mean values were indicated as standard errors of the mean (SEM). Statistical analysis was achieved using Graphpad Prism 8.

## Figures and Tables

**Table 1:** Editing efficiencies induced by ABE8e and ABE7.10 in transgenic plants.

Base Editor	Targets	The Number of Transgenic Plants	The Number of Plants with A-to-G Conversion	A-to-G Editing Efficiency	Heterozygous/Homozygous
PTG-ABE7.10	T1	48	10	20.83%	10/0
	T2	32	5	15.63%	5/0
PTG-ABE8e	T1	42	13	30.95%	11/2
	T2	46	28	60.87%	21/7

**Table 2:** Off-target analysis of transgenic plants transformated with PTG-ABE8e in N. benthamiana.

Target	Off-Target Sites	Putative Off-Target Sequences	Chromosome	Start	End	Strand	The Number of Transgenic Plants	Number of Lines with Off-Targets
T1	1	TAGGTTCTGAAAATTGAGTTTGG	Niben101Scf08162Ctg007	54182	54204	+	42	0
	2	AAATTATTGCAAATTGATTTTGG	Niben101Scf08857Ctg003	22514	22536	+	42	0
	3	AAGTTAATGAAAAATGAGTTAGG	Niben101Scf04437Ctg038	6532	6554	−	42	0
	4	GTACTACTGCAAATTAAGTTAGG	Niben101Scf09870Ctg011	8274	8296	−	42	0
T2	5	TACAAGTTGAACTGGGAGTGGGG	Niben101Scf00726Ctg021	5225	5247	+	46	0
	6	GGCAAATTGAAATGGGAATGTGG	Niben101Scf01483Ctg019	9485	9507	+	46	0
	7	TGCAAAATGTGTTGGAATTGGGG	Niben101Scf04386Ctg039	16421	16443	−	46	0
	8	TGCAAAACGTGTTGGGATTGGGG	Niben101Scf04636Ctg023	11108	11130	+	46	0
	9	TGCAAAATGTGTTGGAATTGGGG	Niben101Scf05346Ctg037	7311	7333	−	46	0

## Data Availability

All data supporting the findings of this study are available in the study, and the NGS data was delivered in the NCBI database. The BioProject ID was PRJNA718450, and the BioSample accessions were ranging from SAMN18533869 to SAMN18533919.

## Supplementary Materials

The following are available online at https://www.mdpi.com/article/10.3390/ijms22115663/s1, Figure S1: Indel frequencies induced by PTG-ABE8e and PTG-ABE7.10 at four target sites based on targeted amplicon deep sequencing results. NT, no-treatment. Figure S2: Indel frequencies induced by ABE8e variants at four target sites based on targeted amplicon deep sequencing results. NT, no-treatment. Figure S3: Sequence of PTG-nCas9. Background color indicated different elements. Figure S4: Sequence of PTG-ABE7.10. Background color indicated different elements. Figure S5: Sequence of PTG-ABE8e. Background color indicated different elements. Figure S6: Sequence of STU-ABE8e. Background color indicated different elements. Figure S7: Sequence of ABE8e. Background color indicated different elements. Table S1: Target sequences used in this study. Table S2: Primer sequences used in this study.
