# Supplementary material for: ABE8e with Polycistronic tRNA-gRNA Expression Cassette Sig-Nificantly Improves Adenine Base Editing Efficiency in Nicotiana benthamiana
Source: Int J Mol Sci. 2021 May 26;22(11):5663. doi: 10.3390/ijms22115663 (PMC8198424; doi:10.3390/ijms22115663)
Supplement: Supplementary file 1 [file ijms-22-05663-s001.zip › ijms-1209566-supplementary.pdf]

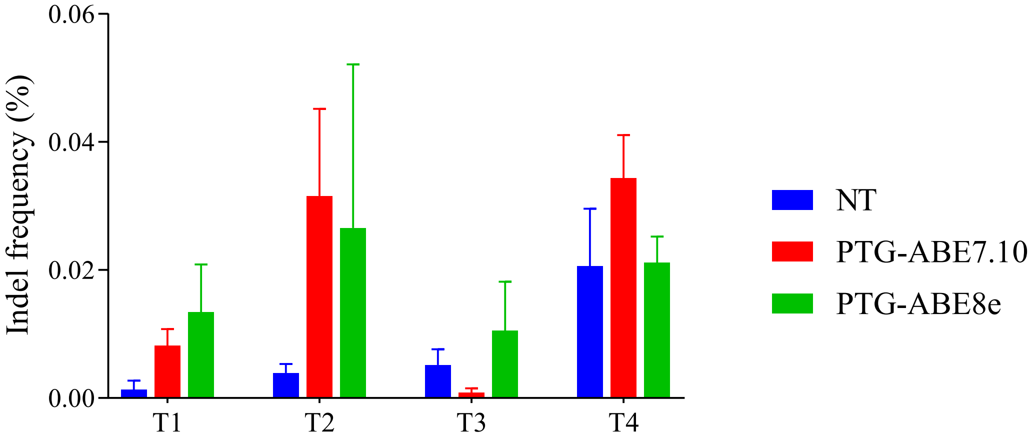

Indel frequency (%)
0.06
0.04
0.02
0.00
T1
T2
T3
T4
NT
PTG-ABE7.10
PTG-ABE8e

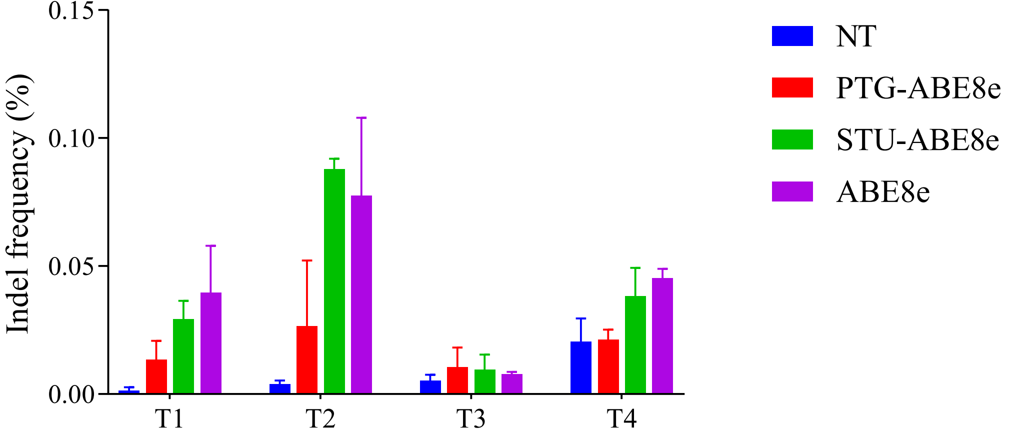

Indel frequency (%)
0.15
0.10
0.05
0.00
T1
T2
T3
T4
NT
PTG-ABE8e
STU-ABE8e
ABE8e

>PTG-nCas9 (35S promoter-linker-32aa linker-nCas9-3xNLS-OCS terminator-AtU6-26-tRNA-BsaI-sgRNA scaffold-polyT)

TGAGACTTTTCAACAAAGGATAATTTCGGGAAACCTCCTCGGATTCCATTGCCCAGCTATCTGTCACTTCATCGAAAGGACAGTAGAAAAGGAAGGTGGCTCCTACAAATGCCATCATTGCGATAAAGGAAAGGC
TATCATTCAAGATCTCTCTGCCGACAGTGGTCCCAAAGATGGACCCCCACCCACGAGGAGCATCGTGGAAAAAGAAGACGTTCCAACCACGTCTTCAAAGCAAGTGGATTGATGTGACATCTCCACTGACGTAA
GGGATGACGCACAATCCCACTATCCTTCGCAAGACCCTTCCTCTATATAAGGAAGTTCATTTCATTTGGAGAGGACACGCTCGAGTATAAGAGCTCTATTTTTACAACAATTACCAACAACAACAAACAACAAACA
ACATTACAATTACATTTACAATTACGGATCCTCCGGCGGATCTTCTGGAGGATCTAGCGGCTCCGAGACACCAGGAACATCCGAATCCGCTACACCAGAGTCTTCTGGAGGATCTAGCGGAGGATCTCTTAAGGA
CAAGAAGTACTCCATCGGCCTCGCCATCGGCACCAACAGCGTCGGCTGGGCGGTGATCACCGACGAGTACAAGGTCCCGTCCAAGAAGTTCAAGGTCCTGGGCAACACCGACCGCCACTCCATCAAGAAGAA
CCTCATCGGCGCCCTCCTCTTCGACTCCGGCGAGACGGCGGAGGCGACCCGCCTCAAGCGCACCGCCCGCCGCCGCTACACCCGCCGCAAGAACCGCATCTGCTACCTCCAGGAGATCTTCTCCAACGAGATG
GCGAAGGTCGACGACTCCTTCTTCCACCGCCTCGAGGAGTCCTTCCTCGTGGAGGAGGACAAGAAGCACGAGCGCCACCCCATCTTCGGCAACATCGTCGACGAGGTCGCCTACCACGAGAAGTACCCCACTA
TCTACCACCTTCGTAAGAAGCTTGTTGACTCTACTGATAAGGCTGATCTTCGTCTCATCTACCTTGCTCTCGCTCACATGATCAAGTTCCGTGGTCACTTCCTTATCGAGGGTGACCTTAACCCTGATAACTCCGACG
TGGACAAGCTCTTCATCCAGCTCGTCCAGACCTACAACCAGCTCTTCGAGGAGAACCCTATCAACGCTTCCGGTGTCGACGCTAAGGCGATCCTTTCCGCTAGGCTCTCCAAGTCCAGGCGTCTCGAGAACCTCA
TCGCCCAGCTCCCTGGTGAGAAGAAGAACGGTCTTTTCGGTAACCTCATCGCTCTCTCCCTCGGTCTGACCCCTAACTTCAAGTCCAACTTCGACCTCGCTGAGGACGCTAAGCTTCAGCTCTCCAAGGATACCTA
CGACGATGATCTCGACAACCTCCTCGCTCAGATTGGAGATCAGTACGCTGATCTCTTCCTTGCTGCTAAGAACCTCTCCGATGCTATCCTCCTTTCGGATATCCTTAGGGTTAACACTGAGATCACTAAGGCTCCTC
TTTCTGCTTCCATGATCAAGCGCTACGACGAGCACCACCAGGACCTCACCCTCCTCAAGGCTCTTGTTCGTCAGCAGCTCCCCGAGAAGTACAAGGAGATCTTCTTCGACCAGTCCAAGAACGGCTACGCCGGTT
ACATTGACGGTGGAGCTAGCCAGGAGGAGTTCTACAAGTTCATCAAGCCAATCCTTGAGAAGATGGATGGTACTGAGGAGCTTCTCGTTAAGCTTAACCGTGAGGACCTCCTTAGGAAGCAGAGGACTTTCGAT
AACGGCTCTATCCCTCACCAGATCCACCTTGGTGAGCTTCACGCCATCCTTCGTAGGCAGGAGGACTTCTACCCTTTCCTCAAGGACAACCGTGAGAAGATCGAGAAGATCCTTACTTTCCGTATTCCTTACTACG
TTGGTCCTCTTGCTCGTGGTAACTCCCGTTTCGCTTGGATGACTAGGAAGTCCGAGGAGACTATCACCCCTTGGAACTTCGAGGAGGTTGTTGACAAGGGTGCTTCCGCCCAGTCCTTCATCGAGCGCATGACCA
ACTTCGACAAGAACCTCCCCAACGAGAAGGTCCTCCCCAAGCACTCCCTCCTCTACGAGTACTTCACGGTCTACAACGAGCTCACCAAGGTCAAGTACGTCACCGAGGGTATGCGCAAGCCTGCCTTCCTCTCC
GGCGAGCAGAAGAAGGCTATCGTTGACCTCCTCTTCAAGACCAACCGCAAGGTCACCGTCAAGCAGCTCAAGGAGGACTACTTCAAGAAGATCGAGTGCTTCGACTCCGTCGAGATCAGCGGCGTTGAGGACC
GTTTCAACGCTTCTCTCGGTACCTACCACGATCTCCTCAAGATCATCAAGGACAAGGACTTCCTCGACAACGAGGAGAACGAGGACATCCTCGAGGACATCGTCCTCACTCTTACTCTCTTCGAGGATAGGGAGA
TGATCGAGGAGAGGCTCAAGACTTACGCTCATCTCTTCGATGACAAGGTTATGAAGCAGCTCAAGCGTCGCCGTTACACCGGTTGGGGTAGGCTCTCCCGCAAGCTCATCAACGGTATCAGGGATAAGCAGAGC
GGCAAGACTATCCTCGACTTCCTCAAGTCTGATGGTTTCGCTAACAGGAACTTCATGCAGCTCATCCACGATGACTCTCTTACCTTCAAGGAGGATATTCAGAAGGCTCAGGTGTCCGGTCAGGGCGACTCTCTCC
ACGAGCACATTGCTAACCTTGCTGGTTCCCCTGCTATCAAGAAGGGCATCCTTCAGACTGTTAAGGTTGTCGATGAGCTTGTCAAGGTTATGGGTCGTCACAAGCCTGAGAACATCGTCATCGAGATGGCTCGTG
AGAACCAGACTACCCAGAAGGGTCAGAAGAACTCGAGGGAGCGCATGAAGAGGATTGAGGAGGGTATCAAGGAGCTTGGTTCTCAGATCCTTAAGGAGCACCCTGTCGAGAACACCCAGCTCCAGAACGAGA
AGCTCTACCTCTACTACCTCCAGAACGGTAGGGATATGTACGTTGACCAGGAGCTCGACATCAACAGGCTTTCTGACTACGACGTCGACCACATTGTTCCTCAGTCTTTCCTTAAGGATGACTCCATCGACAACAA
GGTCCTCACGAGGTCCGACAAGAACAGGGGTAAGTCGGACAACGTCCCTTCCGAGGAGGTTGTCAAGAAGATGAAGAACTACTGGAGGCAGCTTCTCAACGCTAAGCTCATTACCCAGAGGAAGTTCGACAAC
CTCACGAAGGCTGAGAGGGGTGGCCTTTCCGAGCTTGACAAGGCTGGTTTCATCAAGAGGCAGCTTGTTGAGACGAGGCAGATTACCAAGCACGTTGCTCAGATCCTCGATTCTAGGATGAACACCAAGTACGA
CGAGAACGACAAGCTCATCCGCGAGGTCAAGGTGATCACCCTCAAGTCCAAGCTCGTCTCCGACTTCCGCAAGGACTTCCAGTTCTACAAGGTCCGCGAGATCAACAACTACCACCACGCTCACGATGCTTACC
TTAACGCTGTCGTTGGTACCGCTCTTATCAAGAAGTACCCTAAGCTTGAGTCCGAGTTCGTCTACGGTGACTACAAGGTCTACGACGTTCGTAAGATGATCGCCAAGTCCGAGCAGGAGATCGGCAAGGCCACC
GCCAAGTACTTCTTCTACTCCAACATCATGAACTTCTTCAAGACCGAGATCACCCTCGCCAACGGCGAGATCCGCAAGCGCCCTCTTATCGAGACGAACGGTGAGACTGGTGAGATCGTTTGGGACAAGGGTCG
CGACTTCGCTACTGTTCGCAAGGTCCTTTCTATGCCTCAGGTTAACATCGTCAAGAAGACCGAGGTCCAGACCGGTGGCTTCTCCAAGGAGTCTATCCTTCCAAAGAGAAACTCGGACAAGCTCATCGCTAGGAA
GAAGGATTGGGACCCTAAGAAGTACGGTGGTTTCGACTCCCCTACTGTCGCCTACTCCGTCCTCGTGGTCGCCAAGGTGGAGAAGGGTAAGTCGAAGAAGCTCAAGTCCGTCAAGGAGCTCCTCGGCATCACCA
TCATGGAGCGCTCCTCCTTCGAGAAGAACCCGATCGACTTCCTCGAGGCCAAGGGCTACAAGGAGGTCAAGAAGGACCTCATCATCAAGCTCCCCAAGTACTCTCTTTTCGAGCTCGAGAACGGTCGTAAGAGG
ATGCTGGCTTCCGCTGGTGAGCTCCAGAAGGGTAACGAGCTTGCTCTTCCTTCCAAGTACGTGAACTTCCTCTACCTCGCCTCCCACTACGAGAAGCTCAAGGGTTCCCCTGAGGATAACGAGCAGAAGCAGCTC
TTCGTGGAGCAGCACAAGCACTACCTCGACGAGATCATCGAGCAGATCTCCGAGTTCTCCAAGCGCGTCATCCTCGCTGACGCTAACCTCGACAAGGTCCTCTCCGCCTACAACAAGCACCGCGACAAGCCCAT
CCGCGAGCAGGCCGAGAACATCATCCACCTCTTCACGCTCACGAACCTCGGCGCCCCTGCTGCTTTCAAGTACTTCGACACCACCATCGACAGGAAGCGTTACACGTCCACCAAGGAGGTTCTCGACGCTACTC
TCATCCACCAGTCCATCACCGGTCTTTACGAGACTCGTATCGACCTTTCCCAGCTTGGTGGTGATTCCGGCGGCAGCCCAAAGAAGAAGCGGAAGGTGTCTGGAGGTTCTCCTAAGAAAAAGAGAAAAGTGTCC
GGCGGCTCCCCGAAGAAGAAGCGCAAGGTGCTGCTTTAATGAGATATGCGAGACGCCTATGATCGCATGATATTTGCTTTCAATTCTGTTGTGCACGTTGTAAAAAACCTGAGCATGTGTAGCTCAGATCCTTACC
GCCGGTTTCGGTTCATTCTAATGAATATATCACCCGTTACTATCGTATTTTTATGAATAATATTCTCCGTTCAATTTACTGATTGTACCCTACTACTTATATGTACAATATTAAAATGAAAACAATATATTGTGCTGAAT
AGGTTTATAGCGACATCTATGATAGAGCGCCACAATAACAAACAATTGCGTTTTATTATTACAAATCCAATTTTAAAAAAAGCGGCAGAACCGGTCAAACCTAAAAGACTGATTACATAAATCTTATTCAAATTTCA
AAAGTGCCCCAGGGGCTAGTATCTACGACACACCGAGCGGCGAACTAATAACGCTCACTGAAGGGAACTCCGGTTCCCCGCCGGCGCGCATGGGTGAGATTCCTTGAAGTTGAGTATTGGCCGTCCGCTCTAC
CGAAAGTTACGGGCACCATTCAACCCGGTCCAGCACGGCGGCCGGGTAACCGACTTGCTGCCCCGAGAATTATGCAGCATTTTTTTGGTGTATGTGGGCCCCAAATGAAGTGCAGGTCAAACCTTGACAGTGAC
GACAAATCGTTGGGCGGGTCCAGGGCGAATTTTGCGACAACATGTCGAGGCTCAGCAGAAGCTTCGTTGAACAACGGAAACTCGACTTGCCTTCCGCACAATACATCATTTCTTCTTAGCTTTTTTTCTTCTTCTTC
GTTCATACAGTTTTTTTTTGTTTATCAGCTTACATTTTCTTGAACCGTAGCTTTCGTTTTCTTCTTTTTAACTTTCCATTCGGAGTTTTTGTATCTTGTTTCATAGTTTGTCCCAGGATTAGAATGATTAGGCATCGAACCT
TCAAGAATTTGATTGAATAAAACATCTTCATTCTTAAGATATGAAGATAATCTTCAAAAGGCCCCTGGGAATCTGAAAGAAGAGAAGCAGGCCCATTTATATGGGAAAGAACAATAGTATTTCTTATATAGGCCCA
TTTAAGTTGAAAACAATCTTCAAAAGTCCCACATCGCTTAGATAAGAAAACGAAGCTGAGTTTATATACAGCTAGAGTCGAAGTAGTGATTGTCCCTTCGGAACAAAGCACCAGTGGTCTAGTGGTAGAATAGTA
CCCTGCCACGGTACAGACCCGGGTTCGATTCCCGGCTGGTGCAaGAGACCGGTCTCGGTTTCAGAGCTATGCTGGAAACAGCATAGCAAGTTGAAATAAGGCTAGTCCGTTATCAACTTGAAAAAGTGGCACCG
AGTCGGTGCTTTTTT

>PTG-ABE7.10 (35S promoter-linker-TadA-32aa linker-TadA7.10-32aa linker-nCas9-3xNLS-OCS terminator-AtU6-26-tRNA-BsaI-sgRNA scaffold-polyT)

TGAGACTTTTCAACAAAGGATAATTTCGGGAAACCTCCTCGGATTCCATTGCCCAGCTATCTGTCACTTCATCGAAAGGACAGTAGAAAAGGAAGGTGGCTCCTACAAATGCCATCATTGCGATAAAGGAAAGGC
TATCATTCAAGATCTCTCTGCCGACAGTGGTCCCAAAGATGGACCCCCACCCACGAGGAGCATCGTGGAAAAAGAAGACGTTCCAACCACGTCTTCAAAGCAAGTGGATTGATGTGACATCTCCACTGACGTAA
GGGATGACGCACAATCCCACTATCCTTCGCAAGACCCTTCCTCTATATAAGGAAGTTCATTTCATTTGGAGAGGACACGCTCGAGTATAAGAGCTCTATTTTTACAACAATTACCAACAACAACAAACAACAAACA
ACATTACAATTACATTTACAATTACGGATCCATCTGTAGAAGACTGGGCCTTCTTCTGAGCGTTGAAAACTTGCCTCGGCATCCTGAAGAAATAGCAAAGGAGAGCTGCGCACTCGTCTGCAAGAATGCCTTCTGT
GATCTCCACTCTATGATTCATGCCTGGGTAATGAAGAACGTCCATAAGAGAGCCTGCAGCTCCTGTCTTAGCGTTTCTAACGCCGAACACGACCCTGCCAATCCTAGAATGGATCATAGCTCCGGCGCACATAAC
GCAAGGTTCGAAGGTAACGTAGAGTGTGGCGTCGATGAGTCTGTAATTCTGCATAACGAGGCCGCCTTGTCTAAGAGCCATAATCTCTGCGTGAGCTGTTGGATCATGGAGTCCAATGGCTCTATTCCAACCCTC
GCCAATCACCCTGTTATTGAGCACGAGAACAGCTCCAACTGGAACTTCCCTTTCGTCCCTAGCCCTTTTAGCAAGTGTAAGAGCGTGCCTCATCCAATACTCGTGGGAGAACTCCACTTCAGAAGATCCTCCGGA
AGAGCCTCCGCTAGATTCTGGTGTAGCGCTTTCGGATGTTCCTGGTGTCTCGGAGCCGCTAGATCCTCCGGAAGATCCTCCGCTATCTGTAGAAGACTGAGCCTTCTTCTGGGCCTTAATCTCCTGCCTTCTCATCC
TGAAGAAATCGGAAAGAAGAGCAGCGCACTCGTCTGCAAGAATGCCCTCTGTAATCTCCACCCTATGGTTCATGCCTGGATGATGCAACACGTCCATAAGAGATCCGGCGGCTCCTGTTTTAGCGTccctagctccaa
acacgaccctgccaatcctagaatgaatcatagctccggcgcacataacgcaaggttcgagtgtcacgtagagtgtagcgtcaatgagcctgtagttctgcataacgaggccgccTTGTCTGAGAGCCATAATCTCTGCGTGAGCTGTTGGATCATGCCTTCC
AATTGGTCTATTCCAACCCTCGCCGATCACCCTATTATTATGCACAAGAACGGCGCCAACTGGCACTTCTCTTTCGTCCCAAGCTCTCTTAGCAAGTGTAAGAGCGTGCCTCATCCAATACTCGTGAGAGAACTCC
ACCTCGGACATTCCGGCGGATCTTCTGGAGGATCTAGCGGCTCCGAGACACCAGGAACATCCGAATCCGCTACACCAGAGTCTTCTGGAGGATCTAGCGGAGGATCTCTTAAGGACAAGAAGTACTCCATCGGC
CTCGCCATCGGCACCAACAGCGTCGGCTGGGCGGTGATCACCGACGAGTACAAGGTCCCGTCCAAGAAGTTCAAGGTCCTGGGCAACACCGACCGCCACTCCATCAAGAAGAACCTCATCGGCGCCCTCCTCT
TCGACTCCGGCGAGACGGCGGAGGCGACCCGCCTCAAGCGCACCGCCCGCCGCCGCTACACCCGCCGCAAGAACCGCATCTGCTACCTCCAGGAGATCTTCTCCAACGAGATGGCGAAGGTCGACGACTCCT
TCTTCCACCGCCTCGAGGAGTCCTTCCTCGTGGAGGAGGACAAGAAGCACGAGCGCCACCCCATCTTCGGCAACATCGTCGACGAGGTCGCCTACCACGAGAAGTACCCCACTATCTACCACCTTCGTAAGAAG
CTTGTTGACTCTACTGATAAGGCTGATCTTCGTCTCATCTACCTTGCTCTCGCTCACATGATCAAGTTCCGTGGTCACTTCCTTATCGAGGGTGACCTTAACCCTGATAACTCCGACGTGGACAAGCTCTTCATCCA
GCTCGTCCAGACCTACAACCAGCTCTTCGAGGAGAACCCTATCAACGCTTCCGGTGTCGACGCTAAGGCGATCCTTTCCGCTAGGCTCTCCAAGTCCAGGCGTCTCGAGAACCTCATCGCCCAGCTCCCTGGTGA
GAAGAAGAACGGTCTTTTCGGTAACCTCATCGCTCTCTCCCTCGGTCTGACCCCTAACTTCAAGTCCAACTTCGACCTCGCTGAGGACGCTAAGCTTCAGCTCTCCAAGGATACCTACGACGATGATCTCGACAAC
CTCCTCGCTCAGATTGGAGATCAGTACGCTGATCTCTTCCTTGCTGCTAAGAACCTCTCCGATGCTATCCTCCTTTCGGATATCCTTAGGGTTAACACTGAGATCACTAAGGCTCCTCTTTCTGCTTCCATGATCAAG
CGCTACGACGAGCACCACCAGGACCTCACCCTCCTCAAGGCTCTTGTTCGTCAGCAGCTCCCCGAGAAGTACAAGGAGATCTTCTTCGACCAGTCCAAGAACGGCTACGCCGGTTACATTGACGGTGGAGCTAG
CCAGGAGGAGTTCTACAAGTTCATCAAGCCAATCCTTGAGAAGATGGATGGTACTGAGGAGCTTCTCGTTAAGCTTAACCGTGAGGACCTCCTTAGGAAGCAGAGGACTTTCGATAACGGCTCTATCCCTCACCA
GATCCACCTTGGTGAGCTTCACGCCATCCTTCGTAGGCAGGAGGACTTCTACCCTTTCCTCAAGGACAACCGTGAGAAGATCGAGAAGATCCTTACTTTCCGTATTCCTTACTACGTTGGTCCTCTTGCTCGTGGTA
ACTCCCGTTTCGCTTGGATGACTAGGAAGTCCGAGGAGACTATCACCCCTTGGAACTTCGAGGAGGTTGTTGACAAGGGTGCTTCCGCCCAGTCCTTCATCGAGCGCATGACCAACTTCGACAAGAACCTCCCCA
ACGAGAAGGTCCTCCCCAAGCACTCCCTCCTCTACGAGTACTTCACGGTCTACAACGAGCTCACCAAGGTCAAGTACGTCACCGAGGGTATGCGCAAGCCTGCCTTCCTCTCCGGCGAGCAGAAGAAGGCTATC
GTTGACCTCCTCTTCAAGACCAACCGCAAGGTCACCGTCAAGCAGCTCAAGGAGGACTACTTCAAGAAGATCGAGTGCTTCGACTCCGTCGAGATCAGCGGCGTTGAGGACCGTTTCAACGCTTCTCTCGGTAC
CTACCACGATCTCCTCAAGATCATCAAGGACAAGGACTTCCTCGACAACGAGGAGAACGAGGACATCCTCGAGGACATCGTCCTCACTCTTACTCTCTTCGAGGATAGGGAGATGATCGAGGAGAGGCTCAAGA
CTTACGCTCATCTCTTCGATGACAAGGTTATGAAGCAGCTCAAGCGTCGCCGTTACACCGGTTGGGGTAGGCTCTCCCGCAAGCTCATCAACGGTATCAGGGATAAGCAGAGCGGCAAGACTATCCTCGACTTC
CTCAAGTCTGATGGTTTCGCTAACAGGAACTTCATGCAGCTCATCCACGATGACTCTCTTACCTTCAAGGAGGATATTCAGAAGGCTCAGGTGTCCGGTCAGGGCGACTCTCTCCACGAGCACATTGCTAACCTTG
CTGGTTCCCCTGCTATCAAGAAGGGCATCCTTCAGACTGTTAAGGTTGTCGATGAGCTTGTCAAGGTTATGGGTCGTCACAAGCCTGAGAACATCGTCATCGAGATGGCTCGTGAGAACCAGACTACCCAGAAG
GGTCAGAAGAACTCGAGGGAGCGCATGAAGAGGATTGAGGAGGGTATCAAGGAGCTTGGTTCTCAGATCCTTAAGGAGCACCCTGTCGAGAACACCCAGCTCCAGAACGAGAAGCTCTACCTCTACTACCTCC
AGAACGGTAGGGATATGTACGTTGACCAGGAGCTCGACATCAACAGGCTTTCTGACTACGACGTCGACCACATTGTTCCTCAGTCTTTCCTTAAGGATGACTCCATCGACAACAAGGTCCTCACGAGGTCCGACA
AGAACAGGGGTAAGTCGGACAACGTCCCTTCCGAGGAGGTTGTCAAGAAGATGAAGAACTACTGGAGGCAGCTTCTCAACGCTAAGCTCATTACCCAGAGGAAGTTCGACAACCTCACGAAGGCTGAGAGGG
GTGGCCTTTCCGAGCTTGACAAGGCTGGTTTCATCAAGAGGCAGCTTGTTGAGACGAGGCAGATTACCAAGCACGTTGCTCAGATCCTCGATTCTAGGATGAACACCAAGTACGACGAGAACGACAAGCTCATC
CGCGAGGTCAAGGTGATCACCCTCAAGTCCAAGCTCGTCTCCGACTTCCGCAAGGACTTCCAGTTCTACAAGGTCCGCGAGATCAACAACTACCACCACGCTCACGATGCTTACCTTAACGCTGTCGTTGGTACC
GCTCTTATCAAGAAGTACCCTAAGCTTGAGTCCGAGTTCGTCTACGGTGACTACAAGGTCTACGACGTTCGTAAGATGATCGCCAAGTCCGAGCAGGAGATCGGCAAGGCCACCGCCAAGTACTTCTTCTACTCC
AACATCATGAACTTCTTCAAGACCGAGATCACCCTCGCCAACGGCGAGATCCGCAAGCGCCCTCTTATCGAGACGAACGGTGAGACTGGTGAGATCGTTTGGGACAAGGGTCGCGACTTCGCTACTGTTCGCAA
GGTCCTTTCTATGCCTCAGGTTAACATCGTCAAGAAGACCGAGGTCCAGACCGGTGGCTTCTCCAAGGAGTCTATCCTTCCAAAGAGAAACTCGGACAAGCTCATCGCTAGGAAGAAGGATTGGGACCCTAAGA
AGTACGGTGGTTTCGACTCCCCTACTGTCGCCTACTCCGTCCTCGTGGTCGCCAAGGTGGAGAAGGGTAAGTCGAAGAAGCTCAAGTCCGTCAAGGAGCTCCTCGGCATCACCATCATGGAGCGCTCCTCCTTC
GAGAAGAACCCGATCGACTTCCTCGAGGCCAAGGGCTACAAGGAGGTCAAGAAGGACCTCATCATCAAGCTCCCCAAGTACTCTCTTTTCGAGCTCGAGAACGGTCGTAAGAGGATGCTGGCTTCCGCTGGTG
AGCTCCAGAAGGGTAACGAGCTTGCTCTTCCTTCCAAGTACGTGAACTTCCTCTACCTCGCCTCCCACTACGAGAAGCTCAAGGGTTCCCCTGAGGATAACGAGCAGAAGCAGCTCTTCGTGGAGCAGCACAAG
CACTACCTCGACGAGATCATCGAGCAGATCTCCGAGTTCTCCAAGCGCGTCATCCTCGCTGACGCTAACCTCGACAAGGTCCTCTCCGCCTACAACAAGCACCGCGACAAGCCCATCCGCGAGCAGGCCGAGA
ACATCATCCACCTCTTCACGCTCACGAACCTCGGCGCCCCTGCTGCTTTCAAGTACTTCGACACCACCATCGACAGGAAGCGTTACACGTCCACCAAGGAGGTTCTCGACGCTACTCTCATCCACCAGTCCATCA
CCGGTCTTTACGAGACTCGTATCGACCTTTCCCAGCTTGGTGGTGATTCCGGCGGCAGCCCAAAGAAGAAGCGGAAGGTGTCTGGAGGTTCTCCTAAGAAAAAGAGAAAAGTGTCCGGCGGCTCCCCGAAGAA
GAAGCGCAAGGTGCTGCTTTAATGAGATATGCGAGACGCCTATGATCGCATGATATTTGCTTTCAATTCTGTTGTGCACGTTGTAAAAAACCTGAGCATGTGTAGCTCAGATCCTTACCGCCGGTTTCGGTTCATTC
TAATGAATATATCACCCGTTACTATCGTATTTTTATGAATAATATTCTCCGTTCAATTTACTGATTGTACCCTACTACTTATATGTACAATATTAAAATGAAAACAATATATTGTGCTGAATAGGTTTATAGCGACATCT
ATGATAGAGCGCCACAATAACAAACAATTGCGTTTTATTATTACAAATCCAATTTTAAAAAAAGCGGCAGAACCGGTCAAACCTAAAAGACTGATTACATAAATCTTATTCAAATTTCAAAAGTGCCCCAGGGGCT
AGTATCTACGACACACCGAGCGGCGAACTAATAACGCTCACTGAAGGGAACTCCGGTTCCCCGCCGGCGCGCATGGGTGAGATTCCTTGAAGTTGAGTATTGGCCGTCCGCTCTACCGAAAGTTACGGGCACCA
TTCAACCCGGTCCAGCACGGCGGCCGGGTAACCGACTTGCTGCCCCGAGAATTATGCAGCATTTTTTTGGTGTATGTGGGCCCCAAATGAAGTGCAGGTCAAACCTTGACAGTGACGACAAATCGTTGGGCGGG
TCCAGGGCGAATTTTGCGACAACATGTCGAGGCTCAGCAGAAGCTTCGTTGAACAACGGAAACTCGACTTGCCTTCCGCACAATACATCATTTCTTCTTAGCTTTTTTTCTTCTTCTTCGTTCATACAGTTTTTTTTTG
TTTATCAGCTTACATTTTCTTGAACCGTAGCTTTCGTTTTCTTCTTTTTAACTTTCCATTCGGAGTTTTTGTATCTTGTTTCATAGTTTGTCCCAGGATTAGAATGATTAGGCATCGAACCTTCAAGAATTTGATTGAATA
AAACATCTTCATTCTTAAGATATGAAGATAATCTTCAAAAGGCCCCTGGGAATCTGAAAGAAGAGAAGCAGGCCCATTTATATGGGAAAGAACAATAGTATTTCTTATATAGGCCCATTTAAGTTGAAAACAATCT
TCAAAAGTCCCACATCGCTTAGATAAGAAAACGAAGCTGAGTTTATATACAGCTAGAGTCGAAGTAGTGATTGTCCCTTCCGAACAAAGCACCAGTGGTCTAGTGGTAGAATAGTACCCTGCCACGGTACAGAC
CCGGGTTCGATTCCCGGCTGGTGCAaGAGACCGGTCTCGGTTTCAGAGCTATGCTGGAAACAGCATAGCAAGTTGAAATAAGGCTAGTCCGTTATCAACTTGAAAAAGTGGCACCGAGTCGGTGCTTTTTT

>PTG-ABE8e (35S promoter-linker-TadA8e-32aa linker-nCas9-3xNLS-OCS terminator-AtU6-26-tRNA-BsaI-sgRNA scaffold-polyT)

TGAGACTTTTCAACAAAGGATAATTTCGGGAAACCTCCTCGGATTCCATTGCCCAGCTATCTGTCACTTCATCGAAAGGACAGTAGAAAAGGAAGGTGGCTCCTACAAATGCCATCATTGCGATAAAGGAAAGGC
TATCATTCAAGATCTCTCTGCCGACAGTGGTCCCAAAGATGGACCCCCACCCACGAGGAGCATCGTGGAAAAAGAAGACGTTCCAACCACGTCTTCAAAGCAAGTGGATTGATGTGACATCTCCACTGACGTAA
GGGATGACGCACAATCCCACTATCCTTCGCAAGACCCTTCCTCTATATAAGGAAGTTCATTTCATTTGGAGAGGACACGCTCGAGTATAAGAGCTCTATTTTTACAACAATTACCAACAACAACAAACAACAAACA
ACATTACAATTACATTTACAATTACGGATCCatggctcctaagaagaagcggaaggttTCTGAAGTGGAGTTCTCCCACGAGTATTGGATGAGGCACGCTCTTACACTTGCTAAAAGGGCTAGGGACGAAAGGGAAGTTCC
AGTTGGAGCTGTTCTCGTGCTCAATAACAGGGTGATTGGCGAGGGTTGGAATAGAGCCATTGGACTCCATGATCCAACAGCTCACGCAGAGATTATGGCTCTTAGACAAGGCGGCCTCGTTATGCAGAATTACA
GACTCATCGACGCCACACTCTACGTTACCTTCGAACCTTGCGTTATGTGCGCCGGAGCTATGATCCATTCTAGGATTGGCAGGGTCGTGTTCGGCtggAGAAACtctAAGagaGGAGCTGCAGGCTCTCTTATGaatG
TTCTTaatTACCCAGGCATGAATCATAGAGTGGAGATCACAGAAGGCATTCTTGCAGACGAGTGCGCAGCTCTCCTTTGCgatTTCtatAGGATGCCGAGGCAAGTTTTCAACGCTCAGAAGAAGGCCCAGTCTTCTa
ttaatTCCGGCGGATCTTCTGGAGGATCTAGCGGCTCCGAGACACCAGGAACATCCGAATCCGCTACACCAGAGTCTTCTGGAGGATCTAGCGGAGGATCTCTTAAGGACAAGAAGTACTCCATCGGCCTCGCCAT
CGGCACCAACAGCGTCGGCTGGGCGGTGATCACCGACGAGTACAAGGTCCCGTCCAAGAAGTTCAAGGTCCTGGGCAACACCGACCGCCACTCCATCAAGAAGAACCTCATCGGCGCCCTCCTCTTCGACTCC
GGCGAGACGGCGGAGGCGACCCGCCTCAAGCGCACCGCCCGCCGCCGCTACACCCGCCGCAAGAACCGCATCTGCTACCTCCAGGAGATCTTCTCCAACGAGATGGCGAAGGTCGACGACTCCTTCTTCCAC
CGCCTCGAGGAGTCCTTCCTCGTGGAGGAGGACAAGAAGCACGAGCGCCACCCCATCTTCGGCAACATCGTCGACGAGGTCGCCTACCACGAGAAGTACCCCACTATCTACCACCTTCGTAAGAAGCTTGTTGA
CTCTACTGATAAGGCTGATCTTCGTCTCATCTACCTTGCTCTCGCTCACATGATCAAGTTCCGTGGTCACTTCCTTATCGAGGGTGACCTTAACCCTGATAACTCCGACGTGGACAAGCTCTTCATCCAGCTCGTCC
AGACCTACAACCAGCTCTTCGAGGAGAACCCTATCAACGCTTCCGGTGTCGACGCTAAGGCGATCCTTTCCGCTAGGCTCTCCAAGTCCAGGCGTCTCGAGAACCTCATCGCCCAGCTCCCTGGTGAGAAGAAG
AACGGTCTTTTCGGTAACCTCATCGCTCTCTCCCTCGGTCTGACCCCTAACTTCAAGTCCAACTTCGACCTCGCTGAGGACGCTAAGCTTCAGCTCTCCAAGGATACCTACGACGATGATCTCGACAACCTCCTCG
CTCAGATTGGAGATCAGTACGCTGATCTCTTCCTTGCTGCTAAGAACCTCTCCGATGCTATCCTCCTTTCGGATATCCTTAGGGTTAACACTGAGATCACTAAGGCTCCTCTTTCTGCTTCCATGATCAAGCGCTAC
GACGAGCACCACCAGGACCTCACCCTCCTCAAGGCTCTTGTTCGTCAGCAGCTCCCCGAGAAGTACAAGGAGATCTTCTTCGACCAGTCCAAGAACGGCTACGCCGGTTACATTGACGGTGGAGCTAGCCAGG
AGGAGTTCTACAAGTTCATCAAGCCAATCCTTGAGAAGATGGATGGTACTGAGGAGCTTCTCGTTAAGCTTAACCGTGAGGACCTCCTTAGGAAGCAGAGGACTTTCGATAACGGCTCTATCCCTCACCAGATCC
ACCTTGGTGAGCTTCACGCCATCCTTCGTAGGCAGGAGGACTTCTACCCTTTCCTCAAGGACAACCGTGAGAAGATCGAGAAGATCCTTACTTTCCGTATTCCTTACTACGTTGGTCCTCTTGCTCGTGGTAACTCC
CGTTTCGCTTGGATGACTAGGAAGTCCGAGGAGACTATCACCCCTTGGAACTTCGAGGAGGTTGTTGACAAGGGTGCTTCCGCCCAGTCCTTCATCGAGCGCATGACCAACTTCGACAAGAACCTCCCCAACGA
GAAGGTCCTCCCCAAGCACTCCCTCCTCTACGAGTACTTCACGGTCTACAACGAGCTCACCAAGGTCAAGTACGTCACCGAGGGTATGCGCAAGCCTGCCTTCCTCTCCGGCGAGCAGAAGAAGGCTATCGTTG
ACCTCCTCTTCAAGACCAACCGCAAGGTCACCGTCAAGCAGCTCAAGGAGGACTACTTCAAGAAGATCGAGTGCTTCGACTCCGTCGAGATCAGCGGCGTTGAGGACCGTTTCAACGCTTCTCTCGGTACCTAC
CACGATCTCCTCAAGATCATCAAGGACAAGGACTTCCTCGACAACGAGGAGAACGAGGACATCCTCGAGGACATCGTCCTCACTCTTACTCTCTTCGAGGATAGGGAGATGATCGAGGAGAGGCTCAAGACTTA
CGCTCATCTCTTCGATGACAAGGTTATGAAGCAGCTCAAGCGTCGCCGTTACACCGGTTGGGGTAGGCTCTCCCGCAAGCTCATCAACGGTATCAGGGATAAGCAGAGCGGCAAGACTATCCTCGACTTCCTCA
AGTCTGATGGTTTCGCTAACAGGAACTTCATGCAGCTCATCCACGATGACTCTCTTACCTTCAAGGAGGATATTCAGAAGGCTCAGGTGTCCGGTCAGGGCGACTCTCTCCACGAGCACATTGCTAACCTTGCTG
GTTCCCCTGCTATCAAGAAGGGCATCCTTCAGACTGTTAAGGTTGTCGATGAGCTTGTCAAGGTTATGGGTCGTCACAAGCCTGAGAACATCGTCATCGAGATGGCTCGTGAGAACCAGACTACCCAGAAGGGT
CAGAAGAACTCGAGGGAGCGCATGAAGAGGATTGAGGAGGGTATCAAGGAGCTTGGTTCTCAGATCCTTAAGGAGCACCCTGTCGAGAACACCCAGCTCCAGAACGAGAAGCTCTACCTCTACTACCTCCAGA
ACGGTAGGGATATGTACGTTGACCAGGAGCTCGACATCAACAGGCTTTCTGACTACGACGTCGACCACATTGTTCCTCAGTCTTTCCTTAAGGATGACTCCATCGACAACAAGGTCCTCACGAGGTCCGACAAGA
ACAGGGGTAAGTCGGACAACGTCCCTTCCGAGGAGGTTGTCAAGAAGATGAAGAACTACTGGAGGCAGCTTCTCAACGCTAAGCTCATTACCCAGAGGAAGTTCGACAACCTCACGAAGGCTGAGAGGGGTG
GCCTTTCCGAGCTTGACAAGGCTGGTTTCATCAAGAGGCAGCTTGTTGAGACGAGGCAGATTACCAAGCACGTTGCTCAGATCCTCGATTCTAGGATGAACACCAAGTACGACGAGAACGACAAGCTCATCCGC
GAGGTCAAGGTGATCACCCTCAAGTCCAAGCTCGTCTCCGACTTCCGCAAGGACTTCCAGTTCTACAAGGTCCGCGAGATCAACAACTACCACCACGCTCACGATGCTTACCTTAACGCTGTCGTTGGTACCGCT
CTTATCAAGAAGTACCCTAAGCTTGAGTCCGAGTTCGTCTACGGTGACTACAAGGTCTACGACGTTCGTAAGATGATCGCCAAGTCCGAGCAGGAGATCGGCAAGGCCACCGCCAAGTACTTCTTCTACTCCAAC
ATCATGAACTTCTTCAAGACCGAGATCACCCTCGCCAACGGCGAGATCCGCAAGCGCCCTCTTATCGAGACGAACGGTGAGACTGGTGAGATCGTTTGGGACAAGGGTCGCGACTTCGCTACTGTTCGCAAGGT
CCTTTCTATGCCTCAGGTTAACATCGTCAAGAAGACCGAGGTCCAGACCGGTGGCTTCTCCAAGGAGTCTATCCTTCCAAAGAGAAACTCGGACAAGCTCATCGCTAGGAAGAAGGATTGGGACCCTAAGAAGT
ACGGTGGTTTCGACTCCCCTACTGTCGCCTACTCCGTCCTCGTGGTCGCCAAGGTGGAGAAGGGTAAGTCGAAGAAGCTCAAGTCCGTCAAGGAGCTCCTCGGCATCACCATCATGGAGCGCTCCTCCTTCGAG
AAGAACCCGATCGACTTCCTCGAGGCCAAGGGCTACAAGGAGGTCAAGAAGGACCTCATCATCAAGCTCCCCAAGTACTCTCTTTTCGAGCTCGAGAACGGTCGTAAGAGGATGCTGGCTTCCGCTGGTGAGCT
CCAGAAGGGTAACGAGCTTGCTCTTCCTTCCAAGTACGTGAACTTCCTCTACCTCGCCTCCCACTACGAGAAGCTCAAGGGTTCCCCTGAGGATAACGAGCAGAAGCAGCTCTTCGTGGAGCAGCACAAGCACT
ACCTCGACGAGATCATCGAGCAGATCTCCGAGTTCTCCAAGCGCGTCATCCTCGCTGACGCTAACCTCGACAAGGTCCTCTCCGCCTACAACAAGCACCGCGACAAGCCCATCCGCGAGCAGGCCGAGAACAT
CATCCACCTCTTCACGCTCACGAACCTCGGCGCCCCTGCTGCTTTCAAGTACTTCGACACCACCATCGACAGGAAGCGTTACACGTCCACCAAGGAGGTTCTCGACGCTACTCTCATCCACCAGTCCATCACCGG
TCTTTACGAGACTCGTATCGACCTTTCCCAGCTTGGTGGTGATTCCGGCGGCAGCCCAAAGAAGAAGCGGAAGGTGTCTGGAGGTTCTCCTAAGAAAAAGAGAAAAGTGTCCGGCGGCTCCCCGAAGAAGAAG
CGCAAGGTGCTGCTTTAATGAGATATGCGAGACGCCTATGATCGCATGATATTTGCTTTCAATTCTGTTGTGCACGTTGTAAAAAACCTGAGCATGTGTAGCTCAGATCCTTACCGCCGGTTTCGGTTCATTCTAAT
GAATATATCACCCGTTACTATCGTATTTTTATGAATAATATTCTCCGTTCAATTTACTGATTGTACCCTACTACTTATATGTACAATATTAAAATGAAAACAATATATTGTGCTGAATAGGTTTATAGCGACATCTATG
ATAGAGCGCCACAATAACAAACAATTGCGTTTTATTATTACAAATCCAATTTTAAAAAAAGCGGCAGAACCGGTCAAACCTAAAAGACTGATTACATAAATCTTATTCAAATTTCAAAAGTGCCCCAGGGGCTAGT
ATCTACGACACACCGAGCGGCGAACTAATAACGCTCACTGAAGGGAACTCCGGTTCCCCGCCGGCGCGCATGGGTGAGATTCCTTGAAGTTGAGTATTGGCCGTCCGCTCTACCGAAAGTTACGGGCACCATTC
AACCCGGTCCAGCACGGCGGCCGGGTAACCGACTTGCTGCCCCGAGAATTATGCAGCATTTTTTTGGTGTATGTGGGCCCCAAATGAAGTGCAGGTCAAACCTTGACAGTGACGACAAATCGTTGGGCGGGTCC
AGGGCGAATTTTGCGACAACATGTCGAGGCTCAGCAGAAGCTTCGTTGAACAACGGAAACTCGACTTGCCTTCCGCACAATACATCATTTCTTCTTAGCTTTTTTTCTTCTTCTTCGTTCATACAGTTTTTTTTTGTTT
ATCAGCTTACATTTTCTTGAACCGTAGCTTTCGTTTTCTTCTTTTTAACTTTCCATTCGGAGTTTTTGTATCTTGTTTCATAGTTTGTCCCAGGATTAGAATGATTAGGCATCGAACCTTCAAGAATTTGATTGAATAAA
ACATCTTCATTCTTAAGATATGAAGATAATCTTCAAAAGGCCCCTGGGAATCTGAAAGAAGAGAAGCAGGCCCATTTATATGGGAAAGAACAATAGTATTTCTTATATAGGCCCATTTAAGTTGAAAACAATCTTC
AAAAGTCCCACATCGCTTAGATAAGAAAACGAAGCTGAGTTTATATACAGCTAGAGTCGAAGTAGTGATTGTCCCTTCGGAACAAAGCACCAGTGGTCTAGTGGTAGAATAGTACCCTGCCACGGTACAGACCC
GGGTTCGATTCCCGGCTGGTGCAaGAGACCGGTCTCGGTTTCAGAGCTATGCTGGAAACAGCATAGCAAGTTGAAATAAGGCTAGTCCGTTATCAACTTGAAAAAGTGGCACCGAGTCGGTGCTTTTTT)

>STU-ABE8e (35S promoter-linker-TadA8e-32aa linker-nCas9-3xNLS-SV40 poly(A) signal-HH-tRNA-BsaI-sgRNA scaffold-HDV-OCS terminator)

TGAGACTTTTCAACAAAGGATAATTTCGGGAAACCTCCTCGGATTCCATTGCCCAGCTATCTGTCACTTCATCGAAAGGACAGTAGAAAAGGAAGGTGGCTCCTACAAATGCCATCATTGCGATAAAGGAAAGGC
TATCATTCAAGATCTCTCTGCCGACAGTGGTCCCAAAGATGGACCCCCACCCACGAGGAGCATCGTGGAAAAAGAAGACGTTCCAACCACGTCTTCAAAGCAAGTGGATTGATGTGACATCTCCACTGACGTAA
GGGATGACGCACAATCCCACTATCCTTCGCAAGACCCTTCCTCTATATAAGGAAGTTCATTTCATTTGGAGAGGACACGCTCGAGTATAAGAGCTCTATTTTTACAACAATTACCAACAACAACAAACAACAAACA
ACATTACAATTACATTTACAATTACGGATCCatggctcctaagaagaagcggaaggttTCTGAAGTGGAGTTCTCCCACGAGTATTGGATGAGGCACGCTCTTACACTTGCTAAAAGGGCTAGGGACGAAAGGAAGTTCC
AGTTGGAGCTGTTCTCGTGCTCAATAACAGGGTGATTGGCGAGGGTTGGAATAGAGCCATTGGACTCCATGATCCAACAGCTCACGCAGAGATTATGGCTCTTAGACAAGGCGGCCTCGTTATGCAGAATTACA
GACTCATCGACGCCACACTCTACGTTACCTTCGAACCTTGCGTTATGTGCGCCGGAGCTATGATCCATTCTAGGATTGGCAGGGTCGTGTTCGGCtggAGAAACtctAAGagaGGAGCTGCAGGCTCTCTTATGaatG
TTCTTaatTACCCAGGCATGAATCATAGAGTGGAGATCACAGAAGGCATTCTTGCAGACGAGTGCGCAGCTCTCCTTTGCgatTTCtatAGGATGCCGAGGCAAGTTTTCAACGCTCAGAAGAAGGCCCAGTCTTCTa
ttaatTCCGGCGGATCTTCTGGAGGATCTAGCGGCTCCGAGACACCAGGAACATCCGAATCCGCTACACCAGAGTCTTCTGGAGGATCTAGCGGAGGATCTCTTAAGGACAAGAAGTACTCCATCGGCCTCGCCAT
CGGCACCAACAGCGTCGGCTGGGCGGTGATCACCGACGAGTACAAGGTCCCGTCCAAGAAGTTCAAGGTCCTGGGCAACACCGACCGCCACTCCATCAAGAAGAACCTCATCGGCGCCCTCCTCTTCGACTCC
GGCGAGACGGCGGAGGCGACCCGCCTCAAGCGCACCGCCCGCCGCCGCTACACCCGCCGCAAGAACCGCATCTGCTACCTCCAGGAGATCTTCTCCAACGAGATGGCGAAGGTCGACGACTCCTTCTTCCAC
CGCCTCGAGGAGTCCTTCCTCGTGGAGGAGGACAAGAAGCACGAGCGCCACCCCATCTTCGGCAACATCGTCGACGAGGTCGCCTACCACGAGAAGTACCCCACTATCTACCACCTTCGTAAGAAGCTTGTTGA
CTCTACTGATAAGGCTGATCTTCGTCTCATCTACCTTGCTCTCGCTCACATGATCAAGTTCCGTGGTCACTTCCTTATCGAGGGTGACCTTAACCCTGATAACTCCGACGTGGACAAGCTCTTCATCCAGCTCGTCC
AGACCTACAACCAGCTCTTCGAGGAGAACCCTATCAACGCTTCCGGTGTCGACGCTAAGGCGATCCTTTCCGCTAGGCTCTCCAAGTCCAGGCGTCTCGAGAACCTCATCGCCCAGCTCCCTGGTGAGAAGAAG
AACGGTCTTTTCGGTAACCTCATCGCTCTCTCCCTCGGTCTGACCCCTAACTTCAAGTCCAACTTCGACCTCGCTGAGGACGCTAAGCTTCAGCTCTCCAAGGATACCTACGACGATGATCTCGACAACCTCCTCG
CTCAGATTGGAGATCAGTACGCTGATCTCTTCCTTGCTGCTAAGAACCTCTCCGATGCTATCCTCCTTTCGGATATCCTTAGGGTTAACACTGAGATCACTAAGGCTCCTCTTTCTGCTTCCATGATCAAGCGCTAC
GACGAGCACCACCAGGACCTCACCCTCCTCAAGGCTCTTGTTCGTCAGCAGCTCCCCGAGAAGTACAAGGAGATCTTCTTCGACCAGTCCAAGAACGGCTACGCCGGTTACATTGACGGTGGAGCTAGCCAGG
AGGAGTTCTACAAGTTCATCAAGCCAATCCTTGAGAAGATGGATGGTACTGAGGAGCTTCTCGTTAAGCTTAACCGTGAGGACCTCCTTAGGAAGCAGAGGACTTTCGATAACGGCTCTATCCCTCACCAGATCC
ACCTTGGTGAGCTTCACGCCATCCTTCGTAGGCAGGAGGACTTCTACCCTTTCCTCAAGGACAACCGTGAGAAGATCGAGAAGATCCTTACTTTCCGTATTCCTTACTACGTTGGTCCTCTTGCTCGTGGTAACTCC
CGTTTCGCTTGGATGACTAGGAAGTCCGAGGAGACTATCACCCCTTGGAACTTCGAGGAGGTTGTTGACAAGGGTGCTTCCGCCCAGTCCTTCATCGAGCGCATGACCAACTTCGACAAGAACCTCCCCAACGA
GAAGGTCCTCCCCAAGCACTCCCTCCTCTACGAGTACTTCACGGTCTACAACGAGCTCACCAAGGTCAAGTACGTCACCGAGGGTATGCGCAAGCCTGCCTTCCTCTCCGGCGAGCAGAAGAAGGCTATCGTTG
ACCTCCTCTTCAAGACCAACCGCAAGGTCACCGTCAAGCAGCTCAAGGAGGACTACTTCAAGAAGATCGAGTGCTTCGACTCCGTCGAGATCAGCGGCGTTGAGGACCGTTTCAACGCTTCTCTCGGTACCTAC
CACGATCTCCTCAAGATCATCAAGGACAAGGACTTCCTCGACAACGAGGAGAACGAGGACATCCTCGAGGACATCGTCCTCACTCTTACTCTCTTCGAGGATAGGGAGATGATCGAGGAGAGGCTCAAGACTTA
CGCTCATCTCTTCGATGACAAGGTTATGAAGCAGCTCAAGCGTCGCCGTTACACCGGTTGGGGTAGGCTCTCCCGCAAGCTCATCAACGGTATCAGGGATAAGCAGAGCGGCAAGACTATCCTCGACTTCCTCA
AGTCTGATGGTTTCGCTAACAGGAACTTCATGCAGCTCATCCACGATGACTCTCTTACCTTCAAGGAGGATATTCAGAAGGCTCAGGTGTCCGGTCAGGGCGACTCTCTCCACGAGCACATTGCTAACCTTGCTG
GTTCCCCTGCTATCAAGAAGGGCATCCTTCAGACTGTTAAGGTTGTCGATGAGCTTGTCAAGGTTATGGGTCGTCACAAGCCTGAGAACATCGTCATCGAGATGGCTCGTGAGAACCAGACTACCCAGAAGGGT
CAGAAGAACTCGAGGGAGCGCATGAAGAGGATTGAGGAGGGTATCAAGGAGCTTGGTTCTCAGATCCTTAAGGAGCACCCTGTCGAGAACACCCAGCTCCAGAACGAGAAGCTCTACCTCTACTACCTCCAGA
ACGGTAGGGATATGTACGTTGACCAGGAGCTCGACATCAACAGGCTTTCTGACTACGACGTCGACCACATTGTTCCTCAGTCTTTCCTTAAGGATGACTCCATCGACAACAAGGTCCTCACGAGGTCCGACAAGA
ACAGGGGTAAGTCGGACAACGTCCCTTCCGAGGAGGTTGTCAAGAAGATGAAGAACTACTGGAGGCAGCTTCTCAACGCTAAGCTCATTACCCAGAGGAAGTTCGACAACCTCACGAAGGCTGAGAGGGGTG
GCCTTTCCGAGCTTGACAAGGCTGGTTTCATCAAGAGGCAGCTTGTTGAGACGAGGCAGATTACCAAGCACGTTGCTCAGATCCTCGATTCTAGGATGAACACCAAGTACGACGAGAACGACAAGCTCATCCGC
GAGGTCAAGGTGATCACCCTCAAGTCCAAGCTCGTCTCCGACTTCCGCAAGGACTTCCAGTTCTACAAGGTCCGCGAGATCAACAACTACCACCACGCTCACGATGCTTACCTTAACGCTGTCGTTGGTACCGCT
CTTATCAAGAAGTACCCTAAGCTTGAGTCCGAGTTCGTCTACGGTGACTACAAGGTCTACGACGTTCGTAAGATGATCGCCAAGTCCGAGCAGGAGATCGGCAAGGCCACCGCCAAGTACTTCTTCTACTCCAAC
ATCATGAACTTCTTCAAGACCGAGATCACCCTCGCCAACGGCGAGATCCGCAAGCGCCCTCTTATCGAGACGAACGGTGAGACTGGTGAGATCGTTTGGGACAAGGGTCGCGACTTCGCTACTGTTCGCAAGGT
CCTTTCTATGCCTCAGGTTAACATCGTCAAGAAGACCGAGGTCCAGACCGGTGGCTTCTCCAAGGAGTCTATCCTTCCAAAGAGAAACTCGGACAAGCTCATCGCTAGGAAGAAGGATTGGGACCCTAAGAAGT
ACGGTGGTTTCGACTCCCCTACTGTCGCCTACTCCGTCCTCGTGGTCGCCAAGGTGGAGAAGGGTAAGTCGAAGAAGCTCAAGTCCGTCAAGGAGCTCCTCGGCATCACCATCATGGAGCGCTCCTCCTTCGAG
AAGAACCCGATCGACTTCCTCGAGGCCAAGGGCTACAAGGAGGTCAAGAAGGACCTCATCATCAAGCTCCCCAAGTACTCTCTTTTCGAGCTCGAGAACGGTCGTAAGAGGATGCTGGCTTCCGCTGGTGAGCT
CCAGAAGGGTAACGAGCTTGCTCTTCCTTCCAAGTACGTGAACTTCCTCTACCTCGCCTCCCACTACGAGAAGCTCAAGGGTTCCCCTGAGGATAACGAGCAGAAGCAGCTCTTCGTGGAGCAGCACAAGCACT
ACCTCGACGAGATCATCGAGCAGATCTCCGAGTTCTCCAAGCGCGTCATCCTCGCTGACGCTAACCTCGACAAGGTCCTCTCCGCCTACAACAAGCACCGCGACAAGCCCATCCGCGAGCAGGCCGAGAACAT
CATCCACCTCTTCACGCTCACGAACCTCGGCGCCCCTGCTGCTTTCAAGTACTTCGACACCACCATCGACAGGAAGCGTTACACGTCCACCAAGGAGGTTCTCGACGCTACTCTCATCCACCAGTCCATCACCGG
TCTTTACGAGACTCGTATCGACCTTTCCCAGCTTGGTGGTGATTCCGGCGGCAGCCCAAAGAAGAAGCGGAAGGTGTCTGGAGGTTCTCCTAAGAAAAAGAGAAAAGTGTCCGGCGGCTCCCCGAAGAAGAAG
CGCAAGGTGAACTTGTTTATTGCAGCTTATAATGGTTACAAATAAAGCAATAGCATCACAAATTTCACAAATAAAGCATTTTTTTCACTGCATTCTAGTTGTGGTTTGTCCAAACTCATCAATGTATCTTATCATGTCT
GGATttgttctgatgagtccgtgaggacgaaacgagtaagctcgtcAACAAAGCACCAGTGGTCTAGTGGTAGAATAGTACCCTGCCACGGTACAGACCCGGGTTCGATTCCCGGCTGGTGCAaGAGACCGGTCTCGGTTTCAG
AGCTATGCTGGAAACAGCATAGCAAGTTGAAATAAGGCTAGTCCGTTATCAACTTGAAAAAGTGGCACCGAGTCGGTGCCggccggcatggtcccagcctcctcgctggcgccggctgggcaacatgcttcggcatggcgaatgggaCT
GCTTTAATGAGATATGCGAGACGCCTATGATCGCATGATATTTGCTTTCAATTCTGTTGTGCACGTTGTAAAAAACCTGAGCATGTGTAGCTCAGATCCTTACCGCCGGTTTCGGTTCATTCTAATGAATATATCACC
CGTTACTATCGTATTTTTATGAATAATATTCTCCGTTCAATTTACTGATTGTACCCTACTACTTATATGTACAATATTAAAATGAAAACAATATATTGTGCTGAATAGGTTTATAGCGACATCTATGATAGAGCGCCAC
AATAACAAACAATTGCGTTTTATTATTACAAATCCAATTTTAAAAAAAGCGGCAGAACCGGTCAAACCTAAAAGACTGATTACATAAATCTTATTCAAATTTCAAAAGTGCCCCAGGGGCTAGTATCTACGACACA
CCGAGCGGCGAACTAATAACGCTCACTGAAGGGAACTCCGGTTCCCCGCCGGCGCGCATGGGTGAGATTCCTTGAAGTTGAGTATTGGCCGTCCGCTCTACCGAAAGTTACGGGCACCATTCAACCCGGTCCAG
CACGGCGGCCGGGTAACCGACTTGCTGCCCCGAGAATTATGCAGCATTTTTTTGGTGTATGTGGGCCCCAAATGAAGTGCAGGTCAAACCTTGACAGTGACGACAAATCGTTGGGCGGGTCCAGGGCGAATTTT
GCGACAACATGTCGAGGCTCAGCAG

>ABE8e (35S promoter-linker-TadA8e-32aa linker-nCas9-3xNLS-OCS terminator-AtU6-26-BsaI-sgRNA scaffold-polyT)

TGAGACTTTTCAACAAAGGATAATTTCGGGAAACCTCCTCGGATTCCATTGCCCAGCTATCTGTCACTTCATCGAAAGGACAGTAGAAAAGGAAGGTGGCTCCTACAAATGCCATCATTGCGATAAAGGAAAGGC
TATCATTCAAGATCTCTCTGCCGACAGTGGTCCCAAAGATGGACCCCCACCCACGAGGAGCATCGTGGAAAAAGAAGACGTTCCAACCACGTCTTCAAAGCAAGTGGATTGATGTGACATCTCCACTGACGTAA
GGGATGACGCACAATCCCACTATCCTTCGCAAGACCCTTCCTCTATATAAGGAAGTTCATTTCATTTGGAGAGGACACGCTCGAGTATAAGAGCTCTATTTTTACAACAATTACCAACAACAACAAACAACAAACA
ACATTACAATTACATTTACAATTACGGATCCatggctcctaagaagaagcggaaggttTCTGAAGTGGAGTTCTCCCACGAGTATTGGATGAGGCACGCTCTTACACTTGCTAAAAGGGCTAGGGACGAAAGGGAAGTTCC
AGTTGGAGCTGTTCTCGTGCTCAATAACAGGGTGATTGGCGAGGGTTGGAATAGAGCCATTGGACTCCATGATCCAACAGCTCACGCAGAGATTATGGCTCTTAGACAAGGCGGCCTCGTTATGCAGAATTACA
GACTCATCGACGCCACACTCTACGTTACCTTCGAACCTTGCGTTATGTGCGCCGGAGCTATGATCCATTCTAGGATTGGCAGGGTCGTGTTCGGCtggAGAAACtctAAGagaGGAGCTGCAGGCTCTCTTATGaatG
TTCTTaatTACCCAGGCATGAATCATAGAGTGGAGATCACAGAAGGCATTCTTGCAGACGAGTGCGCAGCTCTCCTTTGCgatTTCtatAGGATGCCGAGGCAAGTTTTCAACGCTCAGAAGAAGGCCCAGTCTTCTa
ttaatTCCGGCGGATCTTCTGGAGGATCTAGCGGCTCCGAGACACCAGGAACATCCGAATCCGCTACACCAGAGTCTTCTGGAGGATCTAGCGGAGGATCTCTTAAGGACAAGAAGTACTCCATCGGCCTCGCCAT
CGGCACCAACAGCGTCGGCTGGGCGGTGATCACCGACGAGTACAAGGTCCCGTCCAAGAAGTTCAAGGTCCTGGGCAACACCGACCGCCACTCCATCAAGAAGAACCTCATCGGCGCCCTCCTCTTCGACTCC
GGCGAGACGGCGGAGGCGACCCGCCTCAAGCGCACCGCCCGCCGCCGCTACACCCGCCGCAAGAACCGCATCTGCTACCTCCAGGAGATCTTCTCCAACGAGATGGCGAAGGTCGACGACTCCTTCTTCCAC
CGCCTCGAGGAGTCCTTCCTCGTGGAGGAGGACAAGAAGCACGAGCGCCACCCCATCTTCGGCAACATCGTCGACGAGGTCGCCTACCACGAGAAGTACCCCACTATCTACCACCTTCGTAAGAAGCTTGTTGA
CTCTACTGATAAGGCTGATCTTCGTCTCATCTACCTTGCTCTCGCTCACATGATCAAGTTCCGTGGTCACTTCCTTATCGAGGGTGACCTTAACCCTGATAACTCCGACGTGGACAAGCTCTTCATCCAGCTCGTCC
AGACCTACAACCAGCTCTTCGAGGAGAACCCTATCAACGCTTCCGGTGTCGACGCTAAGGCGATCCTTTCCGCTAGGCTCTCCAAGTCCAGGCGTCTCGAGAACCTCATCGCCCAGCTCCCTGGTGAGAAGAAG
AACGGTCTTTTCGGTAACCTCATCGCTCTCTCCCTCGGTCTGACCCCTAACTTCAAGTCCAACTTCGACCTCGCTGAGGACGCTAAGCTTCAGCTCTCCAAGGATACCTACGACGATGATCTCGACAACCTCCTCG
CTCAGATTGGAGATCAGTACGCTGATCTCTTCCTTGCTGCTAAGAACCTCTCCGATGCTATCCTCCTTTCGGATATCCTTAGGGTTAACACTGAGATCACTAAGGCTCCTCTTTCTGCTTCCATGATCAAGCGCTAC
GACGAGCACCACCAGGACCTCACCCTCCTCAAGGCTCTTGTTCGTCAGCAGCTCCCCGAGAAGTACAAGGAGATCTTCTTCGACCAGTCCAAGAACGGCTACGCCGGTTACATTGACGGTGGAGCTAGCCAGG
AGGAGTTCTACAAGTTCATCAAGCCAATCCTTGAGAAGATGGATGGTACTGAGGAGCTTCTCGTTAAGCTTAACCGTGAGGACCTCCTTAGGAAGCAGAGGACTTTCGATAACGGCTCTATCCCTCACCAGATCC
ACCTTGGTGAGCTTCACGCCATCCTTCGTAGGCAGGAGGACTTCTACCCTTTCCTCAAGGACAACCGTGAGAAGATCGAGAAGATCCTTACTTTCCGTATTCCTTACTACGTTGGTCCTCTTGCTCGTGGTAACTCC
CGTTTCGCTTGGATGACTAGGAAGTCCGAGGAGACTATCACCCCTTGGAACTTCGAGGAGGTTGTTGACAAGGGTGCTTCCGCCCAGTCCTTCATCGAGCGCATGACCAACTTCGACAAGAACCTCCCCAACGA
GAAGGTCCTCCCCAAGCACTCCCTCCTCTACGAGTACTTCACGGTCTACAACGAGCTCACCAAGGTCAAGTACGTCACCGAGGGTATGCGCAAGCCTGCCTTCCTCTCCGGCGAGCAGAAGAAGGCTATCGTTG
ACCTCCTCTTCAAGACCAACCGCAAGGTCACCGTCAAGCAGCTCAAGGAGGACTACTTCAAGAAGATCGAGTGCTTCGACTCCGTCGAGATCAGCGGCGTTGAGGACCGTTTCAACGCTTCTCTCGGTACCTAC
CACGATCTCCTCAAGATCATCAAGGACAAGGACTTCCTCGACAACGAGGAGAACGAGGACATCCTCGAGGACATCGTCCTCACTCTTACTCTCTTCGAGGATAGGGAGATGATCGAGGAGAGGCTCAAGACTTA
CGCTCATCTCTTCGATGACAAGGTTATGAAGCAGCTCAAGCGTCGCCGTTACACCGGTTGGGGTAGGCTCTCCCGCAAGCTCATCAACGGTATCAGGGATAAGCAGAGCGGCAAGACTATCCTCGACTTCCTCA
AGTCTGATGGTTTCGCTAACAGGAACTTCATGCAGCTCATCCACGATGACTCTCTTACCTTCAAGGAGGATATTCAGAAGGCTCAGGTGTCCGGTCAGGGCGACTCTCTCCACGAGCACATTGCTAACCTTGCTG
GTTCCCCTGCTATCAAGAAGGGCATCCTTCAGACTGTTAAGGTTGTCGATGAGCTTGTCAAGGTTATGGGTCGTCACAAGCCTGAGAACATCGTCATCGAGATGGCTCGTGAGAACCAGACTACCCAGAAGGGT
CAGAAGAACTCGAGGGAGCGCATGAAGAGGATTGAGGAGGGTATCAAGGAGCTTGGTTCTCAGATCCTTAAGGAGCACCCTGTCGAGAACACCCAGCTCCAGAACGAGAAGCTCTACCTCTACTACCTCCAGA
ACGGTAGGGATATGTACGTTGACCAGGAGCTCGACATCAACAGGCTTTCTGACTACGACGTCGACCACATTGTTCCTCAGTCTTTCCTTAAGGATGACTCCATCGACAACAAGGTCCTCACGAGGTCCGACAAGA
ACAGGGGTAAGTCGGACAACGTCCCTTCCGAGGAGGTTGTCAAGAAGATGAAGAACTACTGGAGGCAGCTTCTCAACGCTAAGCTCATTACCCAGAGGAAGTTCGACAACCTCACGAAGGCTGAGAGGGGTG
GCCTTTCCGAGCTTGACAAGGCTGGTTTCATCAAGAGGCAGCTTGTTGAGACGAGGCAGATTACCAAGCACGTTGCTCAGATCCTCGATTCTAGGATGAACACCAAGTACGACGAGAACGACAAGCTCATCCGC
GAGGTCAAGGTGATCACCCTCAAGTCCAAGCTCGTCTCCGACTTCCGCAAGGACTTCCAGTTCTACAAGGTCCGCGAGATCAACAACTACCACCACGCTCACGATGCTTACCTTAACGCTGTCGTTGGTACCGCT
CTTATCAAGAAGTACCCTAAGCTTGAGTCCGAGTTCGTCTACGGTGACTACAAGGTCTACGACGTTCGTAAGATGATCGCCAAGTCCGAGCAGGAGATCGGCAAGGCCACCGCCAAGTACTTCTTCTACTCCAAC
ATCATGAACTTCTTCAAGACCGAGATCACCCTCGCCAACGGCGAGATCCGCAAGCGCCCTCTTATCGAGACGAACGGTGAGACTGGTGAGATCGTTTGGGACAAGGGTCGCGACTTCGCTACTGTTCGCAAGGT
CCTTTCTATGCCTCAGGTTAACATCGTCAAGAAGACCGAGGTCCAGACCGGTGGCTTCTCCAAGGAGTCTATCCTTCCAAAGAGAAACTCGGACAAGCTCATCGCTAGGAAGAAGGATTGGGACCCTAAGAAGT
ACGGTGGTTTCGACTCCCCTACTGTCGCCTACTCCGTCCTCGTGGTCGCCAAGGTGGAGAAGGGTAAGTCGAAGAAGCTCAAGTCCGTCAAGGAGCTCCTCGGCATCACCATCATGGAGCGCTCCTCCTTCGAG
AAGAACCCGATCGACTTCCTCGAGGCCAAGGGCTACAAGGAGGTCAAGAAGGACCTCATCATCAAGCTCCCCAAGTACTCTCTTTTCGAGCTCGAGAACGGTCGTAAGAGGATGCTGGCTTCCGCTGGTGAGCT
CCAGAAGGGTAACGAGCTTGCTCTTCCTTCCAAGTACGTGAACTTCCTCTACCTCGCCTCCCACTACGAGAAGCTCAAGGGTTCCCCTGAGGATAACGAGCAGAAGCAGCTCTTCGTGGAGCAGCACAAGCACT
ACCTCGACGAGATCATCGAGCAGATCTCCGAGTTCTCCAAGCGCGTCATCCTCGCTGACGCTAACCTCGACAAGGTCCTCTCCGCCTACAACAAGCACCGCGACAAGCCCATCCGCGAGCAGGCCGAGAACAT
CATCCACCTCTTCACGCTCACGAACCTCGGCGCCCCTGCTGCTTTCAAGTACTTCGACACCACCATCGACAGGAAGCGTTACACGTCCACCAAGGAGGTTCTCGACGCTACTCTCATCCACCAGTCCATCACCGG
TCTTTACGAGACTCGTATCGACCTTTCCCAGCTTGGTGGTGATTCCGGCGGCAGCCCAAAGAAGAAGCGGAAGGTGTCTGGAGGTTCTCCTAAGAAAAAGAGAAAAGTGTCCGGCGGCTCCCCGAAGAAGAAG
CGCAAGGTGCTGCTTTAATGAGATATGCGAGACGCCTATGATCGCATGATATTTGCTTTCAATTCTGTTGTGCACGTTGTAAAAAACCTGAGCATGTGTAGCTCAGATCCTTACCGCCGGTTTCGGTTCATTCTAAT
GAATATATCACCCGTTACTATCGTATTTTTATGAATAATATTCTCCGTTCAATTTACTGATTGTACCCTACTACTTATATGTACAATATTAAAATGAAAACAATATATTGTGCTGAATAGGTTTATAGCGACATCTATG
ATAGAGCGCCACAATAACAAACAATTGCGTTTTATTATTACAAATCCAATTTTAAAAAAAGCGGCAGAACCGGTCAAACCTAAAAGACTGATTACATAAATCTTATTCAAATTTCAAAAGTGCCCCAGGGGCTAGT
ATCTACGACACACCGAGCGGCGAACTAATAACGCTCACTGAAGGGAACTCCGGTTCCCCGCCGGCGCGCATGGGTGAGATTCCTTGAAGTTGAGTATTGGCCGTCCGCTCTACCGAAAGTTACGGGCACCATTC
AACCCGGTCCAGCACGGCGGCCGGGTAACCGACTTGCTGCCCCGAGAATTATGCAGCATTTTTTTGGTGTATGTGGGCCCCAAATGAAGTGCAGGTCAAACCTTGACAGTGACGACAAATCGTTGGGCGGGTCC
AGGGCGAATTTTGCGACAACATGTCGAGGCTCAGCAGAAGCTTCGTTGAACAACGGAAACTCGACTTGCCTTCCGCACAATACATCATTTCTTCTTAGCTTTTTTTCTTCTTCTTCGTTCATACAGTTTTTTTTTGTTT
ATCAGCTTACATTTTCTTGAACCGTAGCTTTCGTTTTCTTCTTTTTAACTTTCCATTCGGAGTTTTTGTATCTTGTTTCATAGTTTGTCCCAGGATTAGAATGATTAGGCATCGAACCTTCAAGAATTTGATTGAATAAA
ACATCTTCATTCTTAAGATATGAAGATAATCTTCAAAAGGCCCCTGGGAATCTGAAAGAAGAGAAGCAGGCCCATTTATATGGGAAAGAACAATAGTATTTCTTATATAGGCCCATTTAAGTTGAAAACAATCTTC
AAAAGTCCCACATCGCTTAGATAAGAAAACGAAGCTGAGTTTATATACAGCTAGAGTCGAAGTAGTGATTGTCCCTTCGGaGAGACCGGTCTCGGTTTCAGAGCTATGCTGGAAACAGCATAGCAAGTTGAAATA
AGGCTAGTCCGTTATCAACTTGAAAAAGTGGCACCGAGTCGGTGCTTTTTT

Table S1 Target sequences used in this study.

Target	Sequences (5'-3')	PAM
T1	GAGTTACTGCAAATTGAGTT	GGG
T2	TGCAAATTGAGTTGGGAGTG	AGG
T3	AAATGATCCGTTAACAGAAG	TGG
T4	GTTCCTAATTCTTTGTTCAG	GGG

Table S2 Primer sequences used in this study.

Primer name	Sequences (5'-3')
gRNA-F	CTTGAAAAAGTGGCACCGAGTCGGTGCAACAAAGCACCAGTGGTCTAGTGGTAGAAT
gRNA-R	ATTCTACCACTAGACCACTGGTGCTTTGTTGCACCGACTCGGTGCCACTTTTTCAAG
Tad7-F	CATTTACAATTACGGATCatggctcctaagaagaagcggaagGTTTCTGAAGTGGAGTTCTCCCACGAGT
Tad7-R	AAGATCCGCCGGAGGATCCATCTGTAGAAGACTGGGCCTTCTTCTGAGCGTTGAAAACT
Tad8e-F	CATTTACAATTACGGATCatggct
Tad8e-R	AAGATCCGCCGGAGGATCCattaatAG
HLW-ABE8E-F	GTTGACCGGTAAGGCGCGCCAAGCTTCGTTGA
HLW-ABE8E-R	GCTAGCTCGAGAGGCGCGCCaaaaaaaaCGAG
PTG-NbPDS-T1-1-F	ggtctctTGCAGAGTTACTGCAAATTGAGTTGTTTCAGAGCTATGCTGGA
PTG-NbPDS-T1-1-R	GGTCTCTaaacAACTCAATTTGCAGTAACTCTGCACCAGCCGGGAATCGA
PTG-NbPDS-T2-1-F	ggtctctTGCATGCAAATTGAGTTGGGAGTGGTTTCAGAGCTATGCTGGA
PTG-NbPDS-T2-1-R	GGTCTCTaaacCACTCCCAACTCAATTTGCATGCACCAGCCGGGAATCGA
PTG-NbPDS-T3-1-F	ggtctctTGCAAAATGATCCGTTAACAGAAGGTTTCAGAGCTATGCTGGA
PTG-NbPDS-T3-1-R	GGTCTCTaaacCTTCTGTTAACGGATCATTTTGCACCAGCCGGGAATCGA
PTG-NbPDS-T4-1-F	ggtctctTGCAGTTCCTAATTCTTTGTTCAGGTTTCAGAGCTATGCTGGA
PTG-NbPDS-T4-1-R	GGTCTCTaaacCTGAACAAAGAATTAGGAACTGCACCAGCCGGGAATCGA
gRNA-R	ggtctccaaaaGCACCGACTCGGTGCCAC
HLW-NbPDS-T1-F	ggtctcgtcggGAGTTACTGCAAATTGAGTTGTTTCAGAGCTATGCTGGA
HLW-NbPDS-T2-F	ggtctcgtcggTGCAAATTGAGTTGGGAGTGGTTTCAGAGCTATGCTGGA
HLW-NbPDS-T3-F	ggtctcgtcggAAATGATCCGTTAACAGAAGGTTTCAGAGCTATGCTGGA
HLW-NbPDS-T4-F	ggtctcgtcggGTTCCTAATTCTTTGTTCAGGTTTCAGAGCTATGCTGGA
F1	TATGGTAGGAAGAGCCAATG
R1	GTATAAACCGCCAAGGAGT
